# Therapeutic potential of hepatocyte-like-cells converted from stem cells from human exfoliated deciduous teeth in fulminant Wilson’s disease

**DOI:** 10.1038/s41598-018-38275-y

**Published:** 2019-02-07

**Authors:** Junko Fujiyoshi, Haruyoshi Yamaza, Soichiro Sonoda, Ratih Yuniartha, Kenji Ihara, Kazuaki Nonaka, Tomoaki Taguchi, Shouichi Ohga, Takayoshi Yamaza

**Affiliations:** 1 Department of Pediatrics, Kyushu University Graduate School of Medical Sciences, Fukuoka, 812-8582 Japan; 2 Department of Pediatric Dentistry, Kyushu University Graduate School of Dental Science, Fukuoka, 812-8582 Japan; 3 Department of Molecular Cell Biology and Oral Anatomy, Kyushu University Graduate School of Dental Science, Fukuoka, 812-8582 Japan; 4 Department of Pediatric Surgery, Kyushu University Graduate School of Medical Sciences, Fukuoka, 812-8582 Japan; 5 Department of Pediatrics, Faculty of Medicine, Oita University, Yuhu, 879-5593 Japan

## Abstract

Wilson’s disease (WD) is an inherited metabolic disease arising from ATPase copper transporting beta gene (*ATP7B*) mutation. Orthotoropic liver transplantation is the only radical treatment of fulminant WD, although appropriate donors are lacking at the onset of emergency. Given the hepatogenic capacity and tissue-integration/reconstruction ability in the liver of stem cells from human exfoliated deciduous teeth (SHED), SHED have been proposed as a source for curing liver diseases. We hypothesized the therapeutic potential of SHED and SHED-converted hepatocyte-like- cells (SHED-Heps) for fulminant WD. SHED and SHED-Heps were transplanted into WD model *Atp7b*-mutated Long-Evans Cinnamon (LEC) rats received copper overloading to induce a lethal fulminant liver failure. Due to the superior copper tolerance via ATP7B, SHED-Hep transplantation gave more prolonged life-span of fulminant LEC rats than SHED transplantation. The integrated ATP7B-expressing SHED-Heps showed more therapeutic effects on to restoring the hepatic dysfunction and tissue damages in the recipient liver than the integrated naïve SHED without ATP7B expression. Moreover, SHED-Heps could reduce copper-induced oxidative stress via ATP7B- independent stanniocalcin 1 secretion in the fulminant LEC rats, suggesting a possible role for paracrine effect of the integrated SHED-Heps. Taken together, SHED-Heps offer a potential of functional restoring, bridging, and preventive approaches for treating fulminant WD.

## Introduction

Wilson’s disease (WD) is an autosomal recessive disorder^1^ and caused by a mutation in the human ATPase copper transporting beta gene (*ATP7B*), which is a critical gene for hepatic copper metabolism^2,3^. Less than 5% of WD cases fall into fulminant liver failure condition^4^. Orthotropic liver transplantation is only a curable treatment of fulminant WD^5^. Almost all patients with fulminant WD rapidly die unless they undergo orthotropic liver transplantation^4^. However, the organ transplant therapy is hard to complete due to the lack of donor livers at the time of diagnosis and the post- transplant problems including life-long requirement for immunosuppressants and neurological complication^6^. Therefore, alternative strategies to rescue, delay, or prevent fulminant WD are urgently required.

Long-Evans Cinnamon (LEC) rats are established in a colony of Long-Evans Agouti (LEA) rats with a spontaneous deletion in the 3′-terminal region of *Atp7B*, which is homologous to the human WD gene, *ATP7B*^7^. LEC rats exhibit WD-like disorders including hepatic copper accumulation and hepatitis. LEC rats also spontaneously develop fulminant hepatitis at the age of 80 to 120 days, and 40–60% of them die under standard diet condition^8^. Dietary copper overloading in LEC rats causes the onset of fulminant hepatitis, resulting in the reduction of lifespan^9^. Cell therapy has been particularly interested in curing WD permanently^10^. Given recent success in allogeneic transplant therapy using primary hepatocytes^11–13^ and *Atp7b*-transfected allogenic mesenchymal stem cells (MSCs)^14^ in the LEC rats, cell therapy is suggested to be effective in rescuing, delaying, and preventing fulminant WD.

Stem cells from human exfoliated deciduous teeth (SHED) are identified as a unique subpopulation of human post-natal MSCs^15^. Concerning the utilities in basic and clinical studies due to the less ethic, cryopreserve ability, high cell expansion, multipotency, and immunosuppressive function^16,17^, suggesting that SHED have been offered a promising strategy in cell therapy. Recent SHED-based cell therapy improves chronic liver cirrhosis in mice due to the *in vivo* significant functions of SHED to tissue-integration, transdifferentiation, and tissue-reconstruction in the recipient liver^18^. Current clinical trials of human MSC- and primary hepatocyte-transplantation provide clinical successes in fulminant hepatic failure^19,20^ and metabolic disorders^21–25^. Human MSCs show a superior therapeutic efficacy compared to human MSC-converted hepatocyte-like cells in drug- induced fulminant hepatic failure model mice^26^. No transplant study using MSCs and MSC-converted hepatocyte-like cells in fulminant hepatic failure of congenital hepatic metabolic diseases such as WD has been reported.

Here, we hypothesized that transplantation of SHED-converted hepatocyte-like cells (SHED-Heps) and SHED may have a potential utility for the control of fulminant WD. In this study, we transplanted SHED-Heps and SHED into LEC rats with fulminant hepatitis under copper overloading and investigated the life-span and the therapeutic efficacy to the fulminant hepatitis in the copper- overloaded LEC rats.

## Results

### Characterization of SHED

Our isolated cells from dental pulp of exfoliated deciduous teeth formed plastic-adherent colonies including spindle-shaped cells and exhibited a highly proliferative potential (Supplementary Fig. S1a–d). The cells expressed CD146, CD105, and CD73, but not CD34, CD45, CD14, CD11b, and human leukocyte antigen (HLA)-class II antigen HLA-DR by flow cytometric analysis (Supplementary Fig. S1e). The cells were differentiated into osteoblasts, chondrocytes, and adipocytes (Supplementary Fig. S1f–h), indicating that our isolated cells were a subpopulation of human MSCs^27^.

### Properties of SHED-Heps

**Figure 1:**
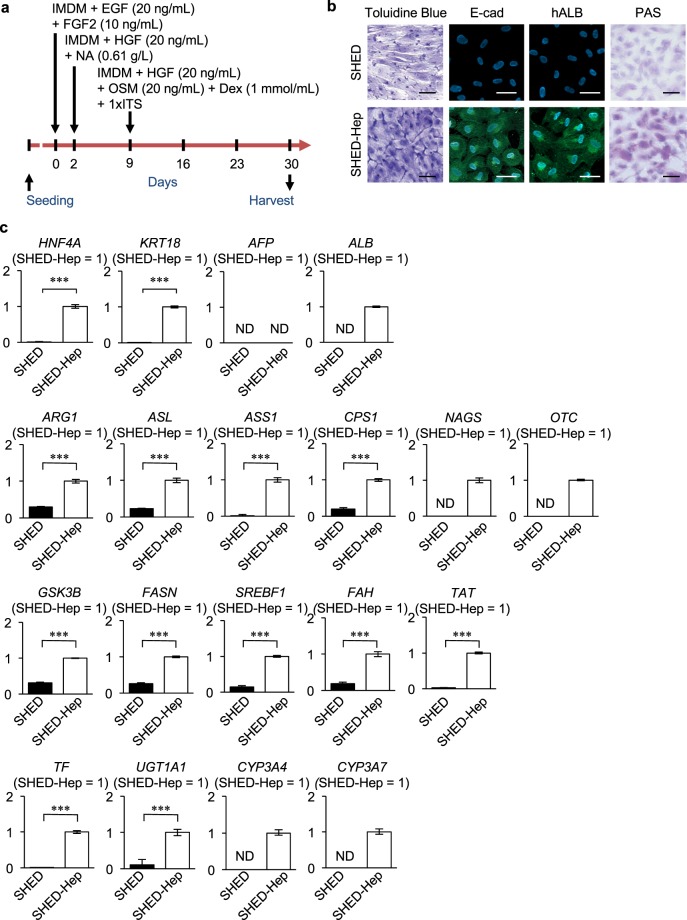
Generation and hepatic characteristics of SHED-Heps. (**a**) A schema of hepatogenic induction of SHED. SHED are stimulated with Iscove’s modified Dulbecco’s media (IMDM) supplemented with epithelial growth factor (EGF; 20 ng/mL) and fibroblast growth factor 2 (FGF2; 10 ng/mL) for 2 days. The cultured cells were then treated under FGF2 (10 ng/mL), hepatocyte growth factor (HGF; 20 ng/mL) and nicotinamide (NA; 0.61 g/L) in IMDM for 7 days, followed by stimulating with oncostatin M (OSM; 20 ng/mL), dexamethasone (Dex; 1 mM) and 1 × ITS premix for 21 days. (**b**) Representative morphological images of SHED- Heps and SHED are taken after treatment with toluidine blue staining, immunofluorescent staining with antibodies against E-cadherin (E-cad) and human albumin (hALB), and periodic acid-Schiff (PAS) staining. Nuclei are stained with 4′,6-diamidino-2-phenylindole (DAPI) in immunofluorescent staining. Bars = 40 μm (**c**) Quantitative reverse transcription polymerase chain reaction (qRT-PCR) analysis shows the expression of hepatocyte-specific genes in SHED-Heps in comparison with SHED. *ALB*, albumin; *ARG1*, arginase 1; *ASL*, argininosuccinate lyase; *ASS1*, argininosuccinate synthase 1; *CPS1*, carbamol-phosphate synthase 1; *CYP3A4*, cytochrome P450 family 3 subfamily A member 4; *CYP3A7*, cytochrome P450 family 3 subfamily A member 7; *FAH*, fumaryl acetoacetate hydrolase; *FASN*, fatty acid synthase; *GSK3B*, glycogen synthase kinase 3 beta; *HNF4A*, hepatocyte nuclear factor 4 alpha; *KRT18*, keratin 18; *NAGS*, N-acetylglutamate synthase; *OTC*, ornithine carbamoyltransferase; *SREBF1*, sterol regulatory element binding transcription factor 1; *TAT*, tyrosine aminotransferase; *TF*, transferrin; *UGT1A1*, UDP glucuronosyltransferase family member A. n = 3 for all groups. ***P < 0.005. ND: no detection. The results are shown as the ratio to each expression in SHED-Heps. Graph bars show the means ± SEM.

**Figure 2:**
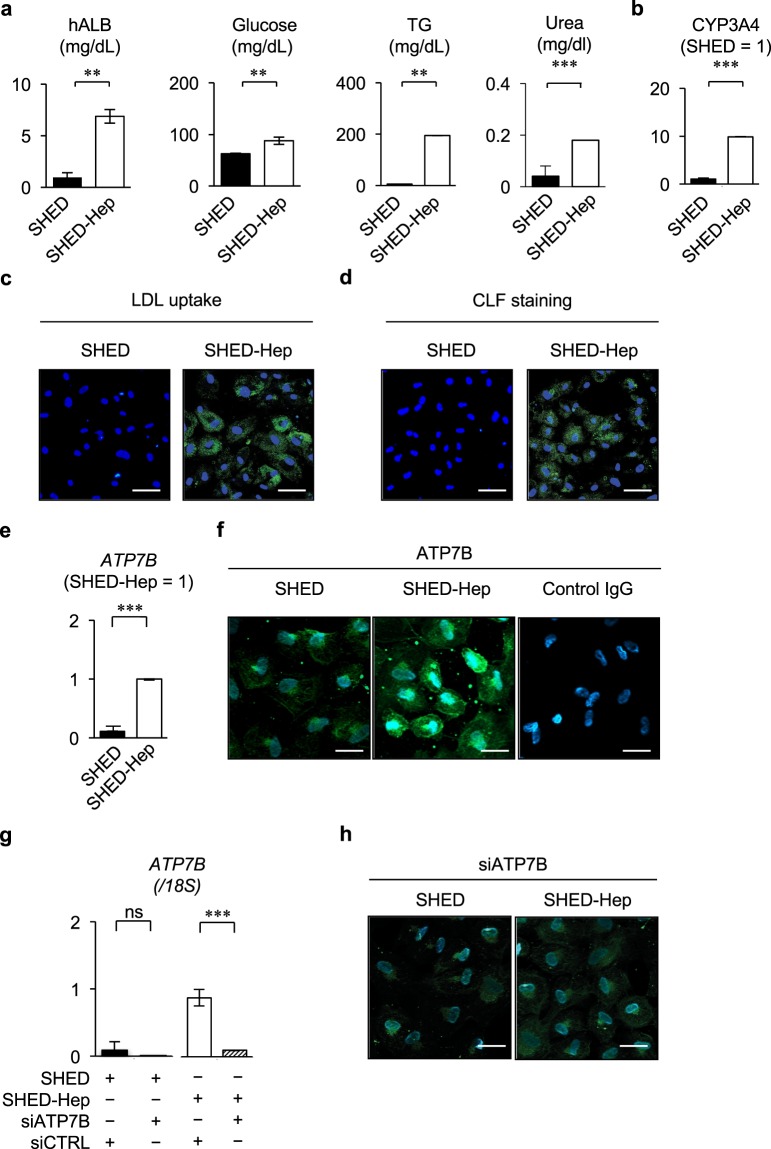
Hepatic functions and ATP7B expression of SHED-Heps. (**a**–**e**) *In vitro* hepatic function assays of SHED-Heps. Culture of SHED-Heps and SHED and measuring of human albumin (hALB), glucose, triglyceride (TG), and urea in the conditioned medium are performed according to the Methods. (**a**) Xenobiotic activity of SHED-Heps and SHED via CYP3A4 is analyzed under dexamethasone stimulation (50 μM). (**b**) Low density lipoprotein (LDL) uptake and bile acid transport are analyzed by DiI-Ac-LDL (**c**) and cholyl-lysyl-fluorescein (CLF) (**d**) staining, respectively. (**e**–**g**) QRT-PCR shows the expression of ATPase copper transporting beta gene (*ATP7B*) in SHED and SHED-Heps. The results are shown as the ratio to the expression in SHED- Heps (**e**). Immunofluorescent staining demonstrates the expression of ATP7B in SHED and SHED- Heps. Control IgG, isotype-matched IgG staining. (**f**) QRT-PCR shows the effect of ATP7B siRNA treatment in SHED and SHED-Heps. The results are shown as the ratio to the expression of 18S ribosomal RNA (*18S*). siATP7B, ATP7B siRNA pre-treatment; siCTRL, scrambled control siRNA pre-treatment (**g**) Immunofluorescent staining shows the effect of ATP7B. siRNA treatment in SHED and SHED-Heps. (**h**) (**a**,**b**,**e**,**g**) n = 3 for all groups. **P < 0.01, ***P < 0.005. Graph bars show the means ± SEM. (**c**,**d**,**f**,**h**) Nuclei are stained with DAPI. Bars = 30 μm (**c**,**d**), 20 μm (**f**,**h**).

Under the present hepatogenic culture condition (Fig. 1a), initial spindle-shaped SHED changed to an epithelial-like polygonal shaped cells (Fig. 1b). The hepatogenically induced cells expressed E- cadherin and human albumin and stored Periodic acid-Schiff staining-positive structures, but the control naïve SHED did not (Fig. 1b). Quantitative reverse transcription polymerase chain reaction (qRT-PCR) analysis demonstrated that the hepatogenically induced SHED expressed several hepatocyte-specific genes (hepatocyte nuclear factor 4 alpha [*HNF4A*], keratin 18 [*KRT18*], albumin [*ALB*], arginase 1 [*ARG1*], argininosuccinate lyase [*ASL*], argininosuccinate synthase 1 [*ASS1*], carbamol-phosphate synthase 1 [*CPS1*], N-acetylglutamate synthase [*NAGS*], ornithine

carbamoyltransferase [*OTC*], glycogen synthase kinase 3 beta [*GSK3B*], fatty acid synthase [*FASN*], sterol regulatory element binding transcription factor 1 [*SREBF1*], fumarylacetoacetate hydrolase [*FAH*], tyrosine aminotransferase [*TAT*], transferrin [*TF*], UDP glucuronosyltransferase family member A1 [*UGT1A1*], cytochrome P450 family 3 subfamily A member 4 [*CYP3A4*], and *CYP3A7*). Meanwhile, naïve SHED showed slight or no expression of these genes (Fig. 1c). Naïve SHED and hepatogenically induced SHED showed no *AFP* expression (Fig. 1c). The hepatogenically induced SHED had abilities to secrete albumin, glucose, triglyceride, and urea into the culture supernatant (Fig. 2a) and expressed a xenobiotic activity via CYP3A4 under dexamethasone stimulation (Fig. 2b). The hepatogenically induced SHED were capable of low-density lipoprotein (LPL) uptake and bile acid transport by by DiI-Ac-LDL and cholyl-lysyl-fluorescein (CLF) staining, respectively (Fig. 2c,d). Meanwhile, naïve SHED exhibited the less activities and capacities of these hepatic functions than the hepatogenically induced SHED (Fig. 2c,d). Moreover, qRT-PCR and immunofluorescent analyses revealed that he hepatogenically induced SHED significantly expressed the WD responsible molecule ATP7B compared to naïve SHED (Fig. 2e,f). Functional knockdown assay using ATP7B siRNA effectively inhibited the expression of *ATP7B* mRNA and ATP7B protein in SHED and SHED-Heps by qRT-PCR and immunofluorescent assays (Fig. 2g,h) Human hepatoblastoma- derived cell line HepG2 cells typically exhibited these hepatic features including hepatocyte-specific gene expression and hepatic functions as seen in the hepatogenically induced SHED (Supplementary Fig. S2). These findings suggested that SHED induced under the present hepatogenic condition express, at least in partially, a feature of hepatocyte-like cells. In this study, we referred the hepatogenically induced cells to SHED-converted hepatocyte-like cells, SHED-Heps.

### Transplantation of SHED-Heps rescues fulminant LEC rats under copper-overloading

**Figure 3:**
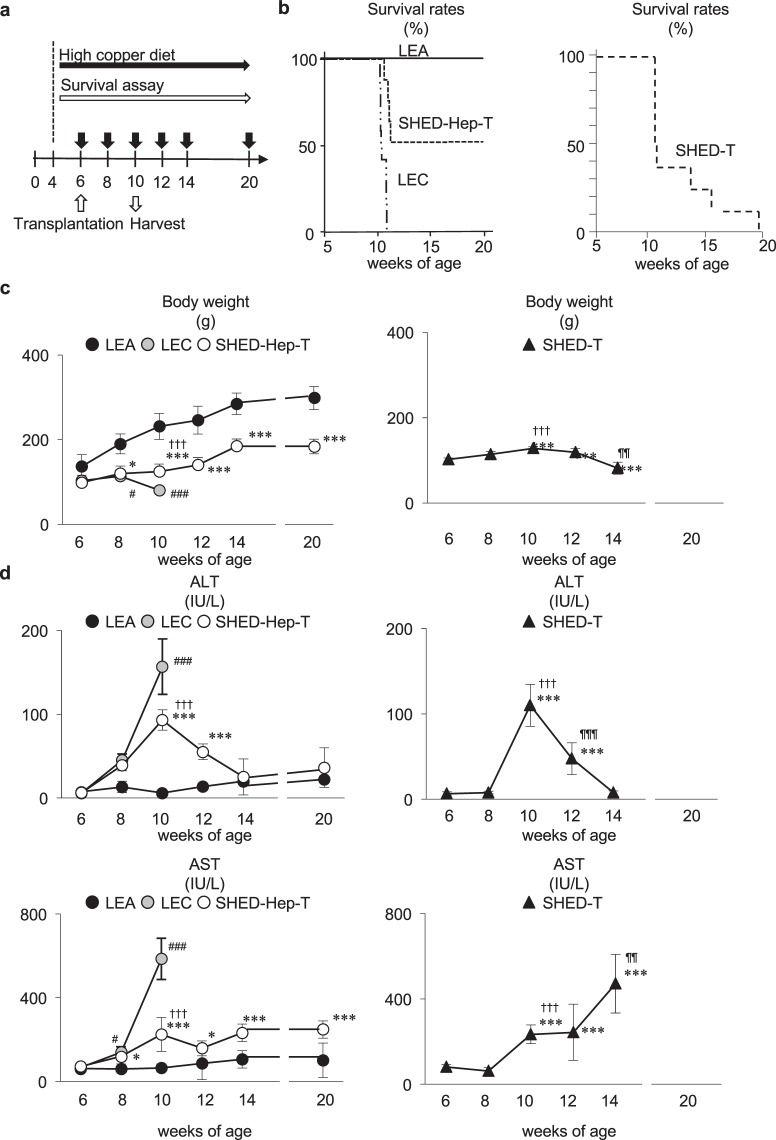
SHED-Hep transplantation prolongs the life span and rescues the hepatic failure in fulminant LEC rats. (**a**) A schema of cell transplantation and assays in fulminant LEC rats. LEC rats receive high copper diets from 4 weeks of the age throughout the animal experiment. SHED and SHED-Heps are transplanted into copper-overloading LEC rats at 6 weeks of the age and are daily inspected the survival. Measurements of body weight and serum markers are performed at the indicated age. Liver tissues are harvested at 10 weeks of the age. (**b**) Survival of animals is assayed by Kaplan-Meier curve. LEA, control LEA rats (n = 7); LEC, fulminant LEC rats (n = 9); SHED-Hep-T, SHED-Hep- transplanted fulminant LEC rats (n = 8); SHED-T, SHED-transplanted fulminant LEC rats (n = 7). (**c**) Body weight of the rats are measured at the indicated age. (**d**) Biochemical assay shows the serum levels of alanine transaminase (ALT) and aspartate aminotransferase (AST) at the indicated age. (**c**,**d**) Black circles, control LEA rats (LEA); ashen circles, fulminant LEC rats (LEC); open circles, SHED- Hep-transplanted fulminant LEC rats (SHED-Hep-T); black triangles, SHED-transplanted fulminant LEC rats (SHED-T). n = 3 for all groups. ^#^P < 0.05 and ^###^P < 0.005 (LEA vs. LEC). *P < 0.05, **P < 0.01, and ***P < 0.005 (LEA vs. SHED-Hep-T or SHED-T). ^†††^P < 0.005 (LEC vs. SHED-Hep-T or SHED-T). ^¶¶¶^P < 0.005 (SHED-Hep-T vs. SHED-T). Graph shows the means ± SD.

**Figure 4:**
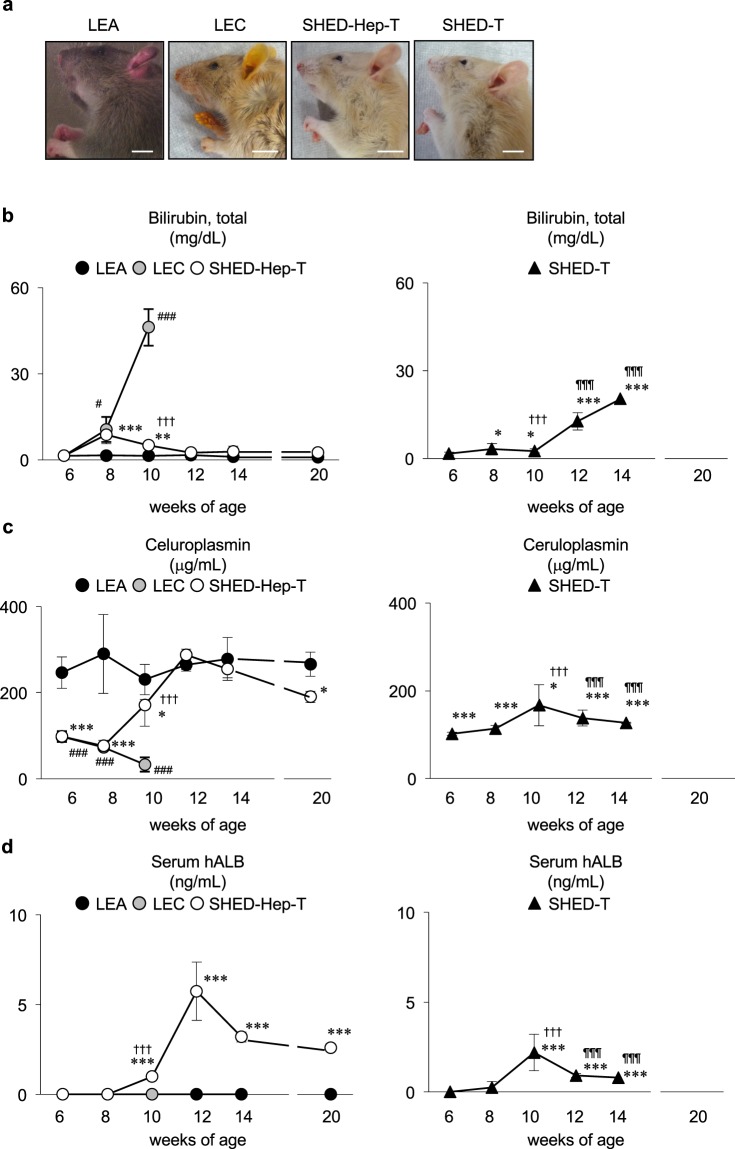
SHED-Hep transplantation rescues the hepatic copper dysfunction in fulminant LEC rats. Rats are treated as indicated in Fig. 3a. (**a**) Representative images of head of the animals are taken at 10 weeks of the age. LEA, control LEA rats; LEC, fulminant LEC rats; SHED-Hep-T, SHED-Hep- transplanted fulminant LEC rats; SHED-T, SHED-transplanted fulminant LEC rats. Bars = 10 mm. (**b**–**d**) Biochemical and immunological assays show the serum levels of total bilirubin (**b**), serum ceruloplasmin (**c**), and serum human albumin (**d**) at the indicated age. n = 3 for all groups. ^#^P < 0.05 and ^###^P < 0.005 (LEA vs. LEC). *P < 0.05, **P < 0.01, and **P < 0.005 (LEA vs. SHED-Hep-T or SHED-T). ^†††^P < 0.005 (LEC vs. SHED-Hep-T or SHED-T). ^¶¶¶^P < 0.005 (SHED-Hep-T vs. SHED-T). Graph shows the means ± SD.

All rats received a high copper diets from 4 weeks of the age and subsequently maintained under the copper overloading until died (Fig. 3a). All copper-overloaded control LEA rats (n = 7) survived without any clinical symptoms throughout the experiment (Figs 3 and 4a–c). Survival assay demonstrated the lethality of copper-overloaded LEC rats by 12 weeks of the age (n = 9; Fig. 3b). All LEC rats died within one week after jaundice became clinically apparent (Fig. 4a). The 6 of 9 LEC rats were died at 10 weeks of the age (Fig. 3b). The copper-overloaded LEC rats at 8 and 10 weeks of the age showed a significant weight loss in comparison with the age-matched LEA rats (Fig. 3c). The copper-overloaded LEC rats exhibited a severe hepatic failure associated with the enhanced serum levels of alanine transaminase (ALT) and aspartate aminotransferase (AST) (Fig. 3d), increased serum level of total bilirubin (Fig. 4b), and reduced serum level of ceruloplasmin (Fig. 4c). These findings indicated that the present copper-overloading protocol was capable of developing a lethal fulminant hepatitis in LEC rats (referred to as “fulminant LEC rats”).

SHED-Heps and SHED (10 × 10^6^ per rat) were intrasplenically transplanted into LEC rats at 6 weeks of the age without any immunosuppressive drug before or after the transplantation (Fig. 3a). The survival assay showed that SHED-Hep-transplantation significantly prolonged the lifespan of the fulminant LEC rats and persisted by 14 weeks after the transplantation (20 weeks of the age) (Fig. 3b). The medians of the survival of the SHED-Hep-transplanted LEC rats (n = 8) was 6 weeks after the transplantation (12 weeks of the age). SHED-Hep-transplantation reversed the significant weight loss and severe hepatic dysfunction in the fulminant LEC rats at 6, 8, 10, 12, 14, and 20 weeks of the age (Figs 3c,d and 4a,b). Meanwhile, all SHED-transplanted fulminant LEC rats (n = 7) slightly prolonged the lifespan and died by 14 weeks after the transplantation (20 weeks of the age) (Fig. 3b). The half maximal survival was yielded by 4 weeks after SHED-transplantation (10 weeks of the age). SHED-transplantation did not show any obvious therapeutic effects on body weight gain and hepatic function throughout the period (Figs 3c,d and 4a–c). On the contrary, all fulminant LEC rats transplanted with human skin fibroblasts (FBs; 10 × 10^6^ per rat; n = 5) died by 6 weeks after the transplantation (12 weeks of the age) (Supplementary Fig. S3a). The FB-transplantation showed no therapeutic effects on body weight gain and hepatic function in fulminant LEC rats (Supplementary Fig. S3b,c). These findings suggested that further FB-transplant assays was not necessary in this study. Further enzyme linked immunosorbent assay demonstrated that human albumin was detected in sera of SHED-Hep-transplanted fulminant LEC rats, but not in those of fulminant LEC or LEA rats, from 4 to 20 weeks post-transplantation (Fig. 4d). Interestingly, low levels of human albumin were detected in SHED-transplanted fulminant LEC rats by 12 weeks after the transplantation (20 weeks of the age) (Fig. 4d).

**Figure 5:**
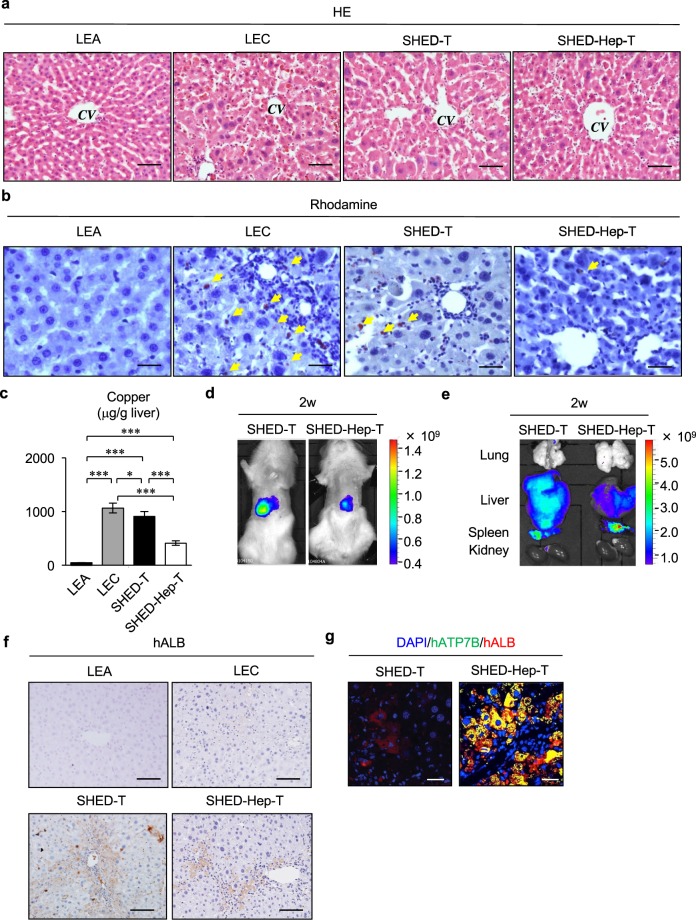
Suppressive effects and integration of transplanted SHED-Heps on copper accumulated hepatic failure in fulminant LEC rats. SHED and SHED-Heps are transplanted in copper-overloaded LEC rats at 6 weeks of the age. (**a**,**b**) Histological assays of liver tissues at 10 weeks of the age. Representative images of liver tissues are analyzed by hematoxylin and eosin staining (HE). *CV*, central vein. (**a**) Copper accumulation is analyzed by Rhodanine staining. Yellow arrows, copper deposition. (**b**,**c**) Biochemical assay shows the copper contents in the liver tissues at 10 weeks of the age. (**d**,**e**) Post-transplant kinetics of DiR- labeled SHED and SHED-Hep cells in fulminant LEC rats after 2 weeks (2w) of SHED- and SHED- Hep-transplantation. *In vivo* tracing shows that DiR labeling is detected in the part of liver of rats. (**d**) *Ex vivo* tracing shows that DiR labeling is detected in liver and spleen, but not in lung and kidney, of rats. (**e**,**f**,**g**) Integration of transplanted SHED- and SHED-Heps in the liver tissues of fulminant LEC rats after 4 weeks of the transplantation. Immunohistochemial assay demonstrates the localization of human albumin (hALB) positive cells in the parenchyma of recipient liver tissues at 10 weeks of the age. Nuclei are stained with hematoxylin. (**f**) Double immunofluorescence shows that localization of human albumin (hALB, red) and human ATP7B (hATP7B, green) in the parenchymal cells of recipient liver tissues of SHED- and SHED-Hep-transplanted fulminant LEC rats. Nuclei are stained with DAPI. (**g**) (**a**–**g**) LEA, control LEA rats; LEC, non-transplanted fulminant LEC rats; SHED-T, SHED-transplanted fulminant LEC rats; SHED-Hep-T, SHED-Hep-transplanted fulminant LEC rats. (**a**,**b**,**f**,**g**) Bars = 50 μm (**a**), 100 μm (**b**,**f**), 30 μm (**g**). (**c**) n = 3 for all groups. Graph bars show the means ± SD. *P < 0.05 and ***P < 0.005.

Subsequent comparative assays were undergone at 10 weeks of the age in the copper-overloaded LEC rats. Histopathological analysis showed that SHED-Hep-transplantation restored the injured parenchymal condition including varied degeneration, enlarged cell bodies and nuclei, fatty microvesicles, and Councilman’s bodies in the liver tissues of copper-overloaded fulminant LEC rats (Fig. 5a). Histochemical and biochemical assays demonstrated that SHED-Hep-transplantation reduced the abundant copper accumulation in the recipient rat livers (Fig. 5b,c). Moreover, qRT- PCR assay demonstrated that SHED-Hep-transplantation markedly reduced the enhanced expression of fulminant hepatitis-associated genes including cyclin dependent kinase inhibitor (*Cdkn1a*), hepatocyte growth factor (*Hgf*), transforming growth factor beta (*Tgfb*), tumor necrosis factor alpha (*Tnfa*), and interleukin 6 (*Il-6*) in the recipient rat livers^28^ (Supplementary Fig. S4). Meanwhile, SHED-transplantation showed a slight therapeutic efficacy to the damaged liver tissues and increased copper accumulation, but not to the enhanced hepatitis-associated genes in fulminant LEC rats (Fig. 5a–c, Supplementary Fig. S5).

### Integration of donor SHED-Heps into the injured recipient liver tissues

*In vivo* imaging assay revealed that the intensity of 1,1′-dioctadecyl-3,3,3′,3′- tetramethylindotricarbocyanine Iodide (DiR) -labeled SHED and SHED-Heps was detected on the skin region corresponding to the liver at the dorsal position after 2 weeks of the infusion (Fig. 5d). *Ex vivo* imaging analysis showed that the recipient livers and spleens were significantly labeled by DiR, but not lungs and kidneys, after 2 weeks of the SHED- and SHED-Hep-transplantation (Fig. 5e).

SHED-transplant rat liver showed a heavier labeling intensity of DiR than SHED-Hep-transplant rat liver (Fig. 5d,e). Immunohistochemical analysis demonstrated that human albumin was detected in the recipient rat liver parenchymal cells after 4 weeks of the SHED-Hep-transplantation, but not in the age-matched control rat livers (Fig. 5f). The replacement frequency of human albumin-positive cells was 11.9 ± 0.8% and 5.6 ± 2.0% (means ± standard deviation [SD]) in the recipient liver of SHED- and SHED-Hep-transplanted LEC rats, respectively. Double-immunofluorescent staining showed that the parenchymal cells of SHED-Hep-transplanted LEC rat liver showed double positive to human albumin and ATP7B, single positive to human albumin alone, and negative to human albumin and ATP7B (Fig. 5g). Meanwhile, no human ATP7B-positive cells were observed in the SHED-transplanted recipient rat livers (Fig. 5g).

### *In vitro* and *in vivo* ATP7B-dependent copper metabolism of SHED-Heps

**Figure 6:**
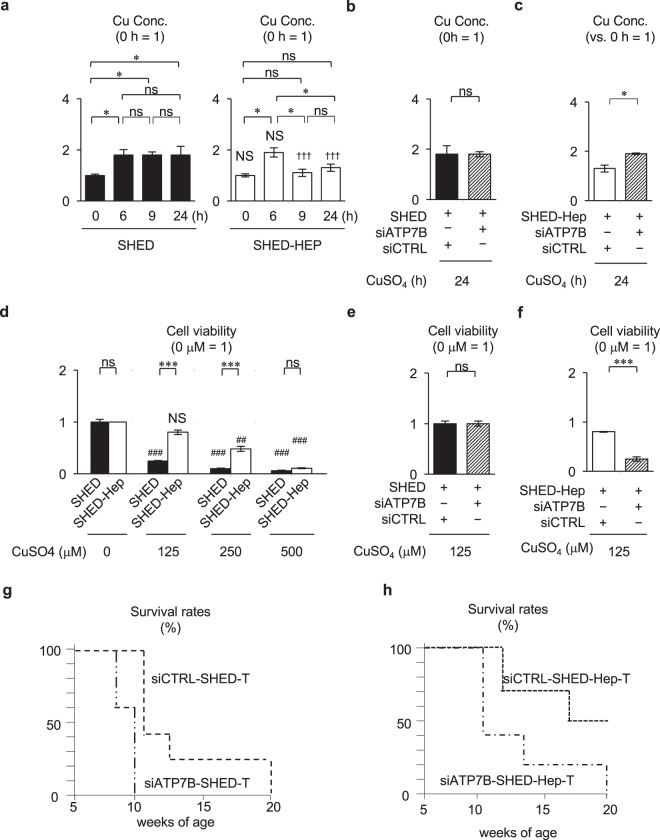
*In vitro* copper metabolism of SHED-Heps. (**a**–**c**) *In vitro* copper metabolism assay in SHED-Heps and SHED via ATP7B. SHED, SHED-Heps, and ATP7B-knock-downed SHED-Heps are cultured for 6 h under CuSO_4_ (600 μM) treatment and are subsequently cultured without CuSO_4_. Intracellular accumulated copper is measured at indicated time. Biochemical assay shows the intracellular copper contents in SHED and SHED-Heps (**a**), ATP7B- knock-downed SHED (**b**), and ATP7B-knock-downed SHED-Heps (**c**). (**d**–**f**) I*n vitro* survival assay in SHED and SHED-Heps via ATP7B under copper stimulation. SHED, SHED-Heps, and ATP7B-knock-downed SHED-Heps are cultured under the different stimulation of CuSO_4_ (0, 125, 250, and 500 μM). The cell viability of the cells is measured after 3 days of the stimulation. Biochemical assay shows the viability of SHED and SHED-Heps (**d**), ATP7B-knock-downed SHED (**e**), and ATP7B- knock-downed SHED-Heps (**f**). (**g**,**h**) ATP7B-siRNA-treated SHED and ATP7B-siRNA-treated SHED-Heps are transplanted into fulminant LEC rats at 6 weeks of the age. The survival of LEC rats transplanted with ATP7B-siRNA-treated SHED (si*ATP7B*-SHED-T; (**g**), n = 5) and ATP7B-siRNA- treated SHED-Heps (si*ATP7B*-SHED-Hep-T; (**h**), n = 5) is assay by Kaplan-Meier curve. (**a**–**f)** The results are shown as the ratio to the copper concentration at 0 h in each group. n = 3 for all groups. *P < 0.05 and ***P < 0.005. ns: no significance. Graph bars show the means ± SEM. (**a**) ^†††^P < 0.005 (vs. SHED at each tipe point). NS: no significance (vs. SHED at each tipe point). (**b**,**c**,**e**,**f**) siATP7B, ATP7B-siRNA treatment; siCTRL, control scrambled siRNA treatment. (**d**) ^###^P < 0.005 (vs. 0 h of each group). NS: no significance (vs. 0 h of each group). (**g**,**h**) CTRL-siRNA-SHED-Hep-T, fulminant LEC rats transplanted with control-siRNA-treated SHED (n = 5).

Under *in vitro* copper (CuSO_4_) exposure condition, SHED-Heps and SHED accumulated copper intracellularly at 6 h after CuSO_4_ exposure (Fig. 6a). After eliminating the copper exposure, in SHED-Heps markedly reduced the intracellularly accumulated copper at 9 and 24 h, but not SHED (Fig. 6a). Functional knockdown assay using ATP7B siRNA demonstrated no differences in the copper excretion or resistivity between ATP7B-siRNA-treated and control siRNA-treated SHED (Fig. 6b). ATP7B siRNA-treated SHED-Heps suppressed the copper excretion, but not control scramble siRNA-treatment (Fig. 6c). *In vitro* cell viability assay demonstrated that the survival of both SHED-Heps and SHED decreased in a CuSO_4_ concentration-dependent manner (Fig. 6d). However, SHED- Heps showed a significantly higher copper resistance than SHED (Fig. 6d). Functional knockdown assay demonstrated no differences in the copper resistivity between ATP7B-siRNA-treated and control siRNA-treated SHED (Fig. 6e). ATP7B siRNA-treatment suppressed the copper resistivity in SHED-Heps, but not control scramble siRNA-treatment (Fig. 6f).

We further infused ATP7B-functionally knock-downed SHED (siATP7B-SHED) and SHED- Heps (siATP7B-SHED-Heps) into the fulminant LEC rats at 6 weeks of the age. The survival of siATP7B-SHED- (n = 5) and siATP7B-SHED-Hep- (n = 5) transplanted fulminant LEC rats was significantly shorter than LEC rats transplanted with control-siRNA-treated SHED (n = 5) (Fig. 6g) and SHED-Heps (n = 5) (Fig. 6h). All rats transplanted with siATP7B-SHED-Heps and siATP7B- SHED-Heps had died by 10 weeks and 14 weeks, respectively, after the transplantation (20 weeks of the age). The siATP7B-SHED-Hep-transplantation did not improve the increased serum levels of ALT, AST, or total bilirubin, the reduced serum levels of ceruloplasmin and the enhanced expression of copper-induced fulminant hepatitis-associated genes in the fulminant LEC rats 4 weeks of the transplantation (Supplementary Figs S6 and S7).

### Effects of SHED-Heps on oxidative injury in fulminant LEC rats

The abnormal accumulated copper causes the enhanced production of reactive oxygen species (ROS) in fulminant hepatitis of LEC rats^28,29^. QRT-PCR and colorimetric analyses showed that SHED-Hep-transplantation suppressed the increased expression of an oxidative stress-associated gene heme oxidase 1 gene (*Hmox1*) and enhanced activity of an oxidative-stress inducer 4-hydroxy-2- deoxyguanosine (4-HNE)^30^ in the fulminant LEC rat livers after 4 weeks of the transplantation (Supplementary Fig. S8a,b). Immunohistochemical analysis showed that SHED-Hep- transplantation reduced the increased number of hepatocytic nuclei positive for 8-hydroxy-2′- deoxyguanosine (8-OHdG), which is a ROS-mediated DNA damage marker^31^, in the fulminant LEC rat livers after 4 weeks of the transplantation (Supplementary Fig. S8c,d).

Next, SHED-Heps were cocultured with HepG2 cells under hydrogen oxide stimulation (25 μM) (Supplementary Fig. S9a). Hydrogen oxide-induced ROS production in HepG2 cells was significantly suppressed in SHED-Heps in a cell number-dependent manner (Supplementary Fig. S9b), suggesting that a paracrine factor from SHED-Heps may participate in the anti-oxidant effect to HepG2. Therefore, we focused on stanniocalcin 1 (STC1), which is antioxidant molecule released from various cells including epithelial cells^32^. STC1 siRNA treatment, which reduced the expression of STC1 mRNA in SHED-Heps (Supplementary Fig. S9c,d), inhibited the SHED-Hep-mediated suppression of ROS production in HepG2 cells under hydrogen oxide stimulation (Supplementary Fig. S9e) The control SHED exhibited a similar paracrine anti-oxidative function via STC1 to SHED-Heps (Supplementary Fig. 9). Transplantation of STC1-siRNA treated SHED-Heps in fulminant LEC rats negatively regulated the SHED-Hep-transplantation-mediated anti-oxidative and anti- hepatitis effects in the recipient livers (Supplementary Fig. S10).

## Discussion

Our *in vitro* findings indicate that a subpopulation of human post-natal MSCs, SHED, can, at least in partially, differentiate into human post-natal MSC-converted hepatocyte-like cells, SHED-Heps, with the sequential stimulation of non-genetic hepatogenic factors, which were evaluated in the previous hepatic induction of human MSCs^33^. SHED-Heps presenting with the specific morphology express specific genes and functional profiles associated with the glycogen storage, production of albumin and urea, and cytochrome P450 activity. In the present study, we demonstrated for the first time that SHED-Heps exhibited a function of copper excretion via ATP7B *in vitro* and *in vivo* compared to SHED, suggesting that SHED-Heps may be a novel candidate for treating WD.

In the present *in vivo* study, using LEC rats with copper-overload-induced lethal fulminant liver failure, we investigated the survival and hepatic copper metabolism governing the success and efficacy of SHED and its converted-hepatocyte-like cells, SHED-Heps, as an alternative treatment for fulminant WD. Our data show that SHED-transplantation had, at least in partially, a potential to treatment fulminant WD. Interestingly, SHED-Hep-transplantation improved the greater rescuing efficacy than SHED-transplantation. Our data showing superior resistance of SHED-Heps to copper stress via ATP7B *in vitro* and *in vivo* indicates that SHED-Heps achieve a feasible donor for fulminant WD treatment. The significance of rescuing lethal fulminant WD model LEC rats suggest that SHED-Hep-transplantation contributes to alternative bridging therapies for WD patients waiting for donor organs. Given the present experimental design of cell transplantation before establishment of fulminant hepatic failure, SHED-Heps might be a useful to the preventive therapy for fulminant WD.

The ultimate goal of therapeutics for WD is the restoration of ATP7B-mediated copper excretion with the regeneration of degenerated liver tissue^10^. The restoration of about 5%-10% of a missing enzyme function in WD gives rise to improve the severe disease phenotype^34^. Although our finding of repopulation frequencies of donor SHED-Heps at 4 weeks post-transplantation is less than 6% of the recipient liver of fulminant LEC rats, the reason why single transplantation of *ATP7B*–expressing SHED-Heps did not rescue all recipient LEC rats from lethal fulminant liver failure has not been fully understood. Spontaneous recruitment capacity of generated matured cells is a critical hurdle for cell replacement therapy^35^. Post-transplant recruitment of SHED-Heps is considered as the maintenance- loss of single transplanted SHED-Heps, because SHED-Heps receive severe cell damage or cell death of by the post-transplant copper-overloading and reduce the cell proliferative capacity under the hepatogenic induction. Multiple infusions of SHED-Heps may contribute to further enhanced therapeutic effects on fulminant WD, as in the case of primary hepatocyte transplantation in fulminant LEC rats^36^.

Several studies on the hepatocyte transplantations in LEC rats showed successful engraftment by recipient-preconditioning surgeries, including radiation, partial hepatectomy, and hepatic ischemia reperfusion to invite the integration of donor cells into the recipient liver^11,12,37^. The surgical preconditioning regimens are hard to be acceptable in the clinical setting for WD treatment. The present non-surgical and copper-induced transplantation model in LEC rats suggests a practical approach to treat WD, especially for the prevention of fulminant WD. However, the present donor cell integration in the recipient liver tissue does not indicate the low immunogenicity of SHED-Heps, because LEC rats have a functional deficiency of T helper cells^38,39^. The immunological safety of donor cells is a critical problem associated with cell therapy for WD^40^. Xenograft study is an issue to overcome for the clinical application. Further studies are required to assess the immunogenicity and immunomodulation of SHED-Heps in SHED-Hep-based therapy for WD.

Interestingly, the present single transplantation of SHED, as well as ATP7B-functionally knock- downed SHED-Heps, in fulminant LEC rats, at least in partially, prolong the life-span. Less engraftment of the human albumin-positive and human ATP7B-negative cells in the recipient liver of SHED-transplanted LEC rats at 4 weeks post-transplantation is also detected. These findings suggest that the therapeutic effects of transplantation of SHED-Heps and SHED in fulminant LEC rats involved mechanisms other than ATP7B-dependent rescuing by transplanted cells. Secretomes derived from SHED ameliorate acute and chronic liver diseases^41,42^, suggesting a benefit of paracrine effects by SHED-Heps and SHED sin the rescue of fulminant WD, even if the therapeutic effects is not the primary attribute and ultimate goal for treating inherited WD. Excessive copper-dependent oxidative stress causes the mitochondrial destruction, DNA damage, and cellular death via ROS in hepatocytes, resulting in tissue failure and adverse effects on the transplanted cells^43,44^. STC1 in mammalian cells is released in paracrine and autocrine fashions and helps to protect against oxidative stress-induced damage in various tissue inflammation^45–47^. Our data showing resistance of SHED- Heps and SHED to oxidative stress via STC1 *in vitro* and *in vivo* suggest a role for the paracrine effects of transplanted SHED-Heps and SHED in fulminant LEC rat.

Taken together, the present findings suggest that SHED-Heps are a novel effective source to rescue and prevent WD with fulminant hepatic failure via restoring the deficient ATB7B function and diminishing copper-induced oxidative stress. Further studies will be necessary in order to enhance the therapeutic efficacy and clinical safety of SHED-Hep-based WD treatment.

## Materials and Methods

### Ethics statement and human subjects

Human deciduous teeth samples were collected as discarded samples from healthy pediatric donors (5–7 years old) in Department of Pediatric Dentistry of Kyushu University Hospital. We obtained written informed consent from each parent on behalf of the child donors. Procedures for handling the human samples were approved by Kyushu University Institutional Review Board for Human Genome/Gene Research (Protocol Number: 393-01). All animal experiments in this study were approved by Institutional Animal Care and Use Committee of Kyushu University (Protocol Number: A21-044-1). All methods were performed in accordance with the relevant guidelines and regulations.

### Animals

WD model LEC and the control LEA rats (female, 4-week-old) were purchased from Institute for Animal Reproduction (Ibaragi, Japan) and were fed water and high copper diet containing larger than 10% of copper (13 mg/kg copper) in a standard chow (MF diet, Oriental Yeast) *ad libitum* under a temperature- and light-controlled environmental condition with a 12-hour light and dark cycle throughout the animal experiment.

### Antibodies

Specific antibodies used in this study were summarized in the Supplementary Table S1.

### Isolation and culture of SHED

Isolation and culture of SHED from remnant dental pulp tissues of exfoliated human deciduous teeth were performed according to previous studies^15^ as described in the Supplementary Methods.

### Hepatogenic differentiation of SHED

P3 SHED were seeded at 0.25 × 10^6^ per dish on human fibronectin-coated 100-mm culture dishes and cultured confluent. SHED were cultured under a hepatogenic condition under serum-free Iscove’s Modified Dulbecco’s Media (IMDM) (Thermo Fisher Scientific, Waltham, MA) containing premixed antibiotics containing 100 U/mL penicillin and 100 μg/mL streptomycin (Nacalai Tesque, Kyoto, Japan). They were initially incubated with epidermal growth factor (EGF; 20 ng/mL; PeproTech, Rocky Hill, NJ) and fibroblast growth factor 2 (FGF2; 10 ng/mL; PeproTech) for 2 days, and then stimulated with FGF2 (10 ng/mL; PeproTech), hepatocyte growth factor (HGF; 20 ng/mL; PeproTech) and nicotinamide (0.61 g/L; Sigma-Aldrich, St. Louis, MO) for 7 days. Finally, the cells were induced under a stimulation of oncostatin M (20 ng/mL; PeproTech), dexamethasone (1 mM; Sigma-Aldrich) and ITS premix (1×; Thermo Fisher Scientific) for 21 days. Each medium was changed twice weekly during the culture.

### Morphological assay of SHED-Heps

Cultured cells were treated with toluidine blue and Periodic acid-Schiff (PAS) staining.

### Immunofluorescence of SHED-Heps

Cultured cells were fixed with 4% paraformaldehyde in PBS at room temperature for 15 min. The cells were incubated with primary antibodies overnight at 4 °C and then treated with Alexa 488- conjugated or Alexa 594-conjugated antibody (Abcam, Cambridge, England) for 60 min. All of the immunofluorescent samples were stained with 4′,6-diamidino-2-phenylindole (1 μg/mL; Dojindo, Kumamoto, Japan). SHED and human hepatocyte cell-lined HepG2 cells (Riken, Tsukuba, Japan) were used as the negative and positive controls, respectively.

### Hepatocyte-specific gene expression assay of SHED-Heps

Expression of human hepatocyte-specific genes in SHED-Heps was analyzed by a quantitative reverse transcript-polymerase chain reaction (qRT-PCR) assay.

### Hepatic biosynthesis assay of SHED-Heps

To measure human albumin and triglyceride in the culture medium, SHED-Heps were cultured for 48 h. To measure urea, cells were cultured for 48 h and incubated with ammonium chloride (5 mM) in Hanks’ balanced salt solution (HBSS) without phenol red for 2 h. To measure glucose, cells were cultured for 24 h in a glucose-free Dulbecco’s modified Eagle medium (Nacalai Tesque) supplemented with sodium pyruvate (2 mM) and sodium lactate (20 mM). Human albumin, triglyceride, urea and glucose in the supernatants were measured using commercially available kits (Supplementary Table S2) according to the manufactures’ instructions. SHED and HepG2 cells (Riken) were used as the negative and positive controls, respectively.

### Cytochrome P450 activity test of SHED-Heps

SHED-Heps were incubated with dexamethasone (50 μM; Sigma-Aldrich). After 24 h, cytochrome P450 3A4 (CYP3A4) activity was measured using a P450-Glo CYP3A4 Kit (Promega, Madison, WN) according to the manufacturer’s instructions. SHED and HepG2 cells (Riken) were used as the negative and positive controls, respectively.

### Hepatic kinetic assay of SHED-Heps

To assess uptake of low-density lipoprotein (LDL), SHED-Heps were incubated with acetylated LDL labeled with 1,1′-dioctadecyl-3,3,3′,3′-tetramethylindo-carbocyanine perchlorate (DiI-Ac-LDL; 10 μg/mL; Biomedical Technologies, Madrid, Spain) for 4 h at 37 °C. To examine transport of bile acid, SHED-Heps were incubated with cholyl-lysyl-fluorescein (CLF; 5 μM; BD Bioscience, Franklin Lake, NJ) in HBSS at 37 °C for 15 min. SHED and HepG2 cells (Riken) were used as the negative and positive controls, respectively.

### *In vivo* monitoring of transplanted cells

SHED-Heps were stained with XenoLight DiR NIR Fluorescent Dye (DiR; 10 μg/mL; Perkin Elmer, Waltham, MA)^48^. The DiR-labeled cells (10 × 10^6^ in 100 μl of PBS) were infused intrasplenically into copper-overloaded LEC rats at 6 weeks of the age. As the control for DiR-labeled-cell monitoring, non-labeled SHED-Heps (10 × 10^6^ in 100 μL of PBS) were infused into the age-matched LEC rats. Ventral images were captured from each animal group after 24 h or 2 weeks under an optical *in vivo* imaging system IVIS Lumina III (Perkin Elmer) and analyzed using living image software (Perkin Elmer).

### *In vitro* copper metabolism assay of SHED-Heps

Expression of human ATP7B: Expression of human *ATP7B* gene and protein in SHED-Heps, SHED and HepG2 cells was analyzed by qRT-PCR assay and immunofluorescence.

Copper excretion assay: SHED-Heps and SHED were stimulated with CuSO_4_ (125 μM; Sigma- Aldrich) for 6 h and incubated without CuSO_4_ additionally for 18 h. The cytosolic concentration of copper was measured using a Metalloassay LS (Metallogenics, Chiba, Japan).

Copper toxic assay: SHED and SHED-Heps were cultured with CuSO_4_ (0, 125, 250, 500 μM). Three days after the stimulation, the cell viability was measured using a Cell Counting Kit-8 (Dojindo).

### SHED-Hep-transplantation, clinical follow-up and survival assay in fulminant LEC rats

SHED-Heps (10 × 10^6^ in 200 μL PBS), SHED (10 × 10^6^ per rat), human fibroblasts (10 × 10^6^ per rat) and PBS (200 μL) were intrasplenically transplanted into copper-overloaded LEC rats at 6 weeks of the age without any immunosuppressant before and after the cell transplantation. The survival and skin color were inspected daily until they died. Measurement of body weight and collection of peripheral blood were carried out at intervals of 2 weeks after the transplantation. Some rats were sacrificed at 10 weeks of the age.

### Histological Preparation

Rat tissues were fixed overnight with 4% paraformaldehyde in PBS at 4 °C. After the samples were dehydrated and were cleaned in xylene, they were embedded in paraffin. Paraffin-embedded samples were cut into 6-μm-thick sections. Some paraffin-embedded sections were treated with hematoxylin and eosin staining or Rhodanine staining^49^. The other sections were used for further staining.

### Immunostaining and immunofluorescence

Paraffin sections were pre-treated to retrieve antigens by microwaving in 0.01 M citrate buffer (pH 8.0). They were then incubated with 0.3% hydrogen peroxide in methanol for 20 min at room temperature to quench endogenous peroxidase activity. The sections were incubated with primary antibodies overnight at 4^o^C. They were treated using a DAKO EnVision kit (Agilent, Santa Clara, CA) and were stained with hematoxylin. For double immunofluorescence, the retrieved paraffin sections were treated with primary antibody, followed by Alexa 488-conjugated antibody (Abcam Cambridge, England). They were then treated with another primary antibody, followed by Alexa 594-conjugated antibody (Abcam). All the immunofluorescent samples were stained with 4′,6-diamidino-2-phenylindole (DAPI; 1 μg/mL; Dojindo). Primary antibodies were listed in the Supplementary Table S1. Immunohistochemical and immunofluorescent controls stained by isotype-matched control antibodies were preliminary confirmed. Five representative images from each animal were randomly selected and were used to measure the percentage of fibrous tissue area or primary antibody-positive area using an Image-J software (National Institutes of Health, Bethesda, MD).

### *In vivo* biochemical and immunological assays

Serum alanine transaminase (ALT), aspartate aminotransferase (AST), total bilirubin, rat ceruloplasmin and human albumin were measured using commercially available kits (Supplementary Table S2) according to the manufactures’ instructions. Liver copper was measured by colorimetric assay using Metalloassay LS (Metallogenics, Chiba, Japan), according to the manufactures’ instructions. Liver 4- hydroxy-2-deoxyguanosine (4-HNE) was measured by ELISA using commercially available kits (OxiSelect HNE Adduct Competitive ELISA kit). Liver gene expression of rat *Cdkn1a*, *Tgfb*, *Tnfa*, *Il6*, *Hgf*, and *Hmox1* was examined by qRT-PCR assay. Rat glyceraldehyde-3-phosphate dehydrogenase was used for normalization.

### *In vitro* anti-oxidative stress assay

ROS production assay: HepG2 cells (3 × 10^5^/well) were incubated in the presence or absence of hydrogen oxide (25 μM) indirectly co-cultured with or without SHED or SHED-Heps (2 × 10^5^, 1 × 10^5^, 0.2 × 10^5^, 0.1 × 10^5^/well) for 4 h. Six hours after the hydrogen oxide treatment, the supernatants were collected and used to measure the ROS using a DCFDA-Cellular Reactive Oxygen Species Detection Assay Kit (Abcam, Cambridge, England).

Human STC-1expession assay: The expression of human ATP7B gene in SHED-Heps was analyzed by a qRT-PCR assay.

### Small interface RNA (siRNA) treatment

SHED and SHED-Heps were treated for 48 h with Lipofectamine RNAiMAX (Thermo Fisher Scientific) mixed with human ATP7B siRNA (20 nM; Santa Cruz Biotechnology, Santa Cruz, CA), human stanniocalcin 1 (STC1) siRNA (20 nM; Santa Cruz Biotechnology, Santa Cruz), and the corresponding control scrambled siRNA (Santa Cruz Biotechnology).

### QRT-PCR assay

To obtain total RNA, cell and tissue samples were treated with TRIzol (Thermo Fisher Scientific). Then the extracts were digested with DNase I (Promega) and were purified using an RNeasy Mini Kit (QIAGEN, Venlo, Netherland). Total RNA was also isolated from the cells and tissues using the SV total RNA isolation system (Promega). The cDNA was prepared by a reverse transcription reaction using a Revertra Ace qPCR kit (TOYOBO, Osaka, Japan). QRT-PCR was performed using target TaqMan probes (Thermo Fisher Scientific) (Supplementary Table S3) and target specific primers (Supplementary Table S4) mixed with EagleTaq Master Mix (Roche, Indianapolis, IN) and SYBR Premix Ex taq II kit (Roche), respectively. Human 18S ribosomal RNA was used for normalization.

### Statistical analysis

Statistical results were expressed as the mean ± standard error of the mean (SEM) or mean ± standard deviation (SD), at least, triplicate determinations. Comparisons between two groups were analyzed by independent two-tailed Student’s t-tests. Multiple group comparison was analyzed by One-way repeated measures analysis of variance (ANOVA) followed by the Tukey post hoc test. Kaplan Meier analyses and Kruskal-Wallis test were used for survival assay. The values of P < 0.05 were considered to be significant. All of the statics were performed using a PRISM 6 software (GraphPad, Software, La Jolla, CA).

## Supplementary information

Supplementary information

## Data Availability

All data generated or analysed during this study are included in this published article and its Supplementary files.
