# Supplementary material for: Therapeutic potential of hepatocyte-like-cells converted from stem cells from human exfoliated deciduous teeth in fulminant Wilson’s disease
Source: Sci Rep. 2019 Feb 7;9:1535. doi: 10.1038/s41598-018-38275-y (PMC6367569; doi:10.1038/s41598-018-38275-y)
Supplement: Supplementary file 1 — Supplementary information [file 41598_2018_38275_MOESM1_ESM.pdf]

Supplementary Information

Therapeutic potential of hepatocyte-like-cells converted from stem cells from human exfoliated deciduous teeth in Wilson's disease

Junko Fujiyoshi, Haruyoshi Yamaza, Soichiro Sonoda, Ratih Yuniartha, Kenji Ihara, Kazuaki Nonaka, Tomoaki Taguchi, Shouichi Ohga, Takayoshi Yamaza

Supplementary Methods

Isolation and culture of stem cells from exfoliated deciduous teeth (SHED)

Dental pulp tissues from human deciduous teeth were digested with 0.3% collagenase type I (Worthington Biochemicals, Lakewood, NJ) and 0.4% dispase II (Sanko Junyaku Co., Ltd., Tokyo, Japan) for 60 min at 37°C. The obtained cells were seeded on culture flasks. After three hours of the seeding, the cultures were washed with sterilized phosphate-buffered saline (PBS). The adherent cells were grown with a growth medium containing of 15% fetal bovine serum (Equitech-Bio, Kerrville, TX), 100 μM L-ascorbic acid 2-phosphate (Wako Pure Chemicals, Osaka, Japan), 2 mM L-glutamine (Nacalai Tesque, Kyoto, Japan), and premixed antibiotics containing 100 U/mL penicillin and 100 μg/ml streptomycin (Nacalai Tesque) in alpha Modification of Eagle's Medium (Thermo Fisher Scientific, Waltham, MA). The isolated cells formed attached colonies consisting of spindle-shaped cells on plastic culture flasks. The adherent colony-forming cells were passaged and were sub-cultured. The medium was changed twice a week. The passaged 3 (P3) cells were determined the characteristics as MSCs as described below.

Characterization of SHED

The characterization of SHED was determined by previous standard[1,2] according to previous reports.[3,4]

Adherent colony-forming capacity: Adherent colony-forming capacity of SHED was evaluated by colony-forming unit fibroblasts assay.[5] Isolated cells were seeded and were cultured in the growth medium for 16 days. The flasks were treated with 4% paraformaldehyde and 0.1% toluidine blue in PBS for 18 h. Attached colonies containing >50 cells were observed under a microscopy.

Cell proliferation capacity: P3 SHED were cultured in the growth medium for 2 days and were examined using a bromodeoxyuridine incorporation assay kit (Thermo Fisher Scientific).

Population doubling assay: Cells were seeded on T-75 culture flasks. When the cells reached at sub-confluent condition, the cells were passed. These steps were repeated until the cells lost dividing capability. Finally, the population doubling score was calculated.

Telomerase activity test: SHED (P3, $5x10^3$) were used for measuring the telomerase activity by a telomere repeat amplification protocol assay using a quantitative telomerase detection kit (Allied Biotech, Inc., Ijamsville, MD). The average starting quantity of fluorescence units was used to compare the telomerase activity among the samples.

Expression of cell surface markers: Flow cytometric analysis of SHED (P3) was performed to examine the expression of human CD146, CD105, CD73, CD45, CD34, CD14, CD11b, and human leukocyte antigen DR (HLA-DR). The number (percentage) of positive cells was determined on a FACSVerse flow cytometer (BD Bioscience, Franklin Lake, NJ) and were analyzed using FACSuite software (BD Bioscience) in comparison with the corresponding control cells stained with corresponding isotype-matched antibody in which a false-positive rate of less than 1% was accepted.

In vitro multipotent assay:

(1) *in vitro* osteogenic induction assay. P3 SHED were grown in the growth medium until confluent condition and induced with an osteogenic medium supplemented with 1.8 mM potassium dihydrogen phosphate (Sigma-Aldrich, St. Louis, MO) and 10 nM dexamethasone (Sigma-Aldrich). The osteogenic medium was changed twice a week. The cultures were also stained with 1% Alizarin Red-S at 4 weeks post induction and analyzed mineralized nodule formation.

(2) *in vitro* chondrogenic induction assay. SHED (P3; 0.2×10^6) were aggregated and cultured with Dulbecco's modified Eagle's medium (Thermo Fisher Scientific) supplemented with 15%

FBS (Equitech-Bio), 2 mM L-glutamine (Nacalai Tesque), 100 μM L-ascorbate-2-phosphate (Wako Pure Chemicals), 2 mM sodium pyruvate (Nacalai Tesque), 1% insulin-transferrin-selenium mixture (ITS) (Thermo Fisher Scientific), 100 nM dexamethasone (Sigma-Aldrich) and 10 ng/mL transforming growth factor β_1 (TGFβ_1) (PeproTech, Rocky Hill, NJ). The chondrogenic medium was changed twice a week. The aggregated tissues were stained with 1% Alcian blue.

(3) *in vitro* adipogenic induction assay. P3 SHED were cultured until the confluent condition and induced in an adipogenic medium supplemented with 500 μM isobutyl-methylxanthine (Sigma-Aldrich), 60 μM indomethacin (Sigma-Aldrich), 0.5 μM hydrocortisone (Sigma-Aldrich) and 10 μM insulin (Sigma-Aldrich). The cultures were stained with 0.3% Oil red O (Sigma-Aldrich) at 6 week induction.

References

1 Dominici, M. *et al.* Minimal criteria for defining multipotent mesenchymal stromal cells. The International Society for Cellular Therapy position statement. *Cytotherapy (Oxford, England)* **8**, 315-317 (2006).

2 Yamaza, T., Sonoda, S., Tomoda, E. & Tanaka, Y. Properties and Possibilities of Human Dental Pulp-Derived Stem Cells. *Arch Stem Cell Res* **2**, 1012 (2015).

3 Yamaza, T. *et al.* Immunomodulatory properties of stem cells from human exfoliated deciduous teeth. *Stem Cell. Res. Ther.* **1** (2010).

4 Ma, L. *et al.* Cryopreserved dental pulp tissues of exfoliated deciduous teeth is a feasible stem cell resource for regenerative medicine. *PLoS One* **7** (2012).

5 Friedenstein, A. J. *et al.* Precursors for fibroblasts in different populations of hematopoietic cells as detected by the in vitro colony assay method. *Exp. Hematol.* **2**, 83-92 (1974).

Supplementary Table S1. The list of specific antibodies.

Names of antibodies	Types of antibodies	Names of Suppliers
anti-albumin, human, antibody	purified	Agilent (Santa Clara, CA)
anti-ATP7B antibody	purified	Abcam (Cambridge, England)
anti-cadherin, E antibody	purified	Abcam (Cambridge, England)
anti-CD11b antibody	R-PE-conjugated	Biolegend (San Diego, CA)
anti-CD14 antibody	R-PE -conjugated	Biolegend (San Diego, CA)
anti-CD34 antibody	R-PE-conjugated	Biolegend (San Diego, CA)
anti-CD45 antibody	R-PE-conjugated	Biolegend (San Diego, CA)
anti-CD73 antibody	R-PE-conjugated	Biolegend (San Diego, CA)
anti-CD105 antibody	R-PE-conjugated	Biolegend (San Diego, CA)
anti-CD146 antibody	R-PE-conjugated	Biolegend (San Diego, CA)
anti-HLA-DR antibody	R-PE-conjugated	Biolegend (San Diego, CA)
anti-8OHdG antibody	purified	Abcam (Cambridge, England))

HepPar1: human hepatocyte paraffin 1
HLA-DR: human leukocyte antigen DR
8OHdG: 8-hydroxy-2'-deoxyguanosine
R-PE: R-phycoerythrin

Supplementary Table S2. The list of commercially available kits for colorimetric assays and ELISA.

Names of kits	Names of Suppliers
AssayMAX Human Albumin ELISA Kit	AssayPro (St. Charles, MO)
AssayMAX Rat Ceruloplasmin ELISA kit	AssayPro (St. Charles, MO)
Glucose CII-test	Wako Pure Chemicals (Tokyo, Japan)
QuantiChrom Bilirubin Assay Kit	BioAssay Systems (Hayward, CA)
QuantiChrom Urea Assay Kit	BioAssay Systems (Hayward, CA)
Transaminase CII-Test Kit	Wako Pure Chemicals (Tokyo, Japan)
Triglyceride E-test	Wako Pure Chemicals (Tokyo, Japan)

Supplementary Table S3. List of TaqMan probes used for qRT-PCR

Names of genes	Gene assay ID Numbers
AFP	Hs00173490_m1
ALB	Hs00910225_m1
ARG1	Hs00982833_m1
ASL	Hs00902699_m1
ASS1	Hs01597989_g1
ATP7B	Hs00163739_m1
CPS1	Hs00157048_m1
CYP3A4	Hs00604506_m1
CYP3A7	Hs00426361_m1
FAH	Hs00164611_m1
FASN	Hs01005622_m1
GSK3B	Hs01047719_m1
HNF4A	Hs01378672_m1
KRT18	Hs02827483_g1
NAGS	Hs00400246_m1
OTC	Hs00166892_m1
SREBF1	Hs01088691_m1
STC1	Hs00174970_m1
TAT	Hs00356930_m1
TF	Hs01067777_m1
UGT1A1	Hs02511055_m1
18S ribosomal RNA	Hs99999901_s1

Supplementary Table S4. The list of primer pairs for qRT-PCR.

Cdkn1a (GenBank accession no. U24174)
sense: 5'- CTTGCACTCTGGTGTCTCACG -3' (nucleotide 431-451)
antisense: 5'- ATCGGCGCTTGGAGTGATAG -3' (nucleotide 534-553)

Hgf (GenBank accession no. NM_017017)
sense: 5'- GACATTCCTCAGTGTTCAGAAGTTG -3' (nucleotide 749-773)
antisense: 5'- TGCCTGATTCTGTGTGATCCA -3' (nucleotide 813-833)

Hmox1 (GenBank accession no. NM_012580)
sense: 5'- AGAGGCTAAGACCGCCTTCC -3' (nucleotide 719-738)
antisense: 5'- AGGCCTCTGGCGAAGAAAC -3' (nucleotide 818-836)

Il6 (GenBank accession no. E02522)
sense: 5'-TCCTACCCCAACTTCCAATGCTC-3' (nucleotide 532-554)
antisense: 5'-TTGGATGGTCTTGGTCCTTAGCC-3' (nucleotide 588-610)

Tgfb (GenBank accession no. X52498.1)
sense: 5'- TGGCGTTACCTTGGTAACC -3' (nucleotide 947-965)
antisense: 5'- GGTGTTGAGCCCTTTCCAG -3' (nucleotide 1205-1223)

Tnfa (GenBank accession no. X66539)
sense: 5'-AAATGGGCTCCCTCTCATCAGTTC-3' (nucleotide 195-218)
antisense: 5'-TCTGCTTGGTGGTTTGCTACGAC-3' (nucleotide 283-305)

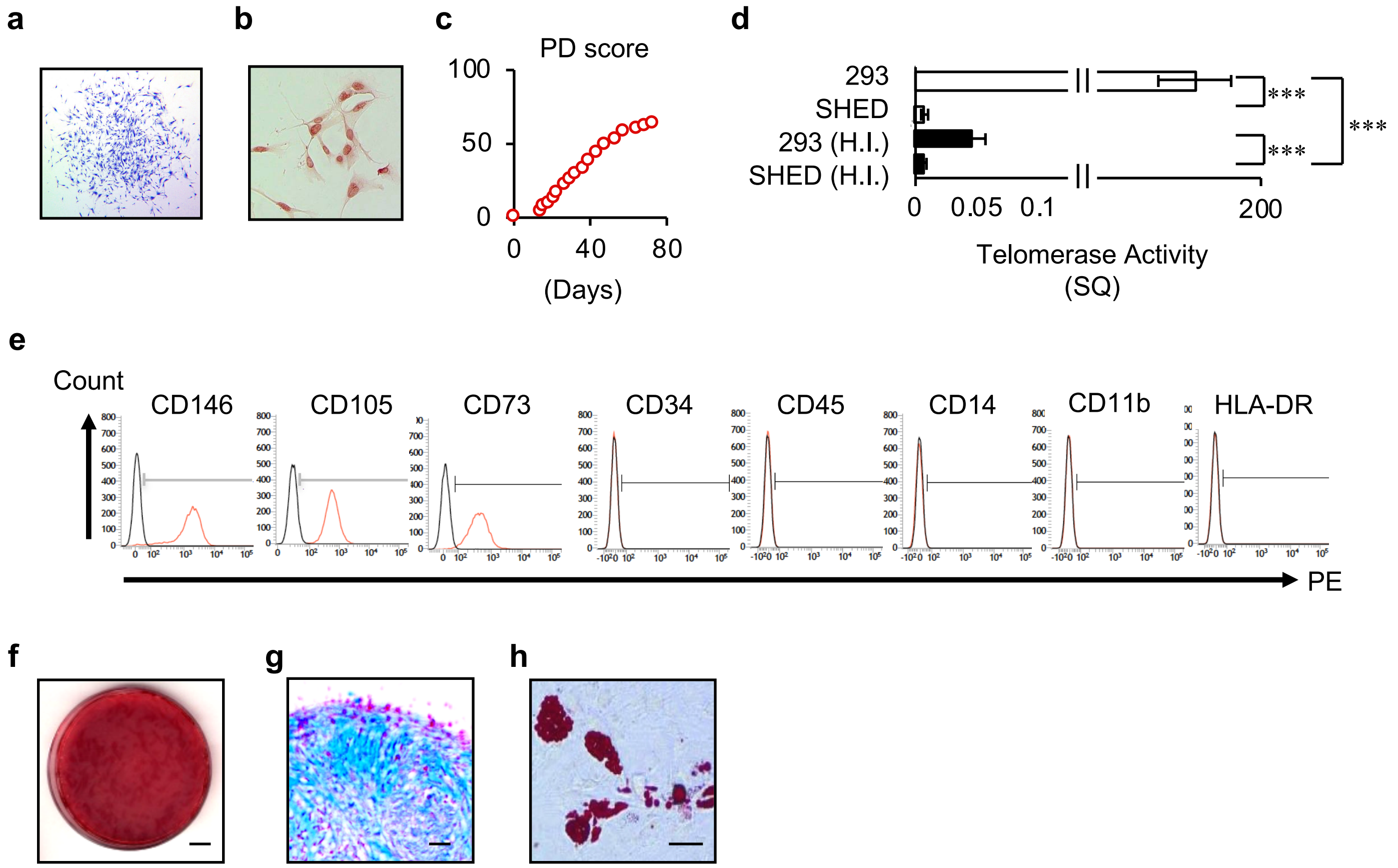

Supplementary Figure S1. Characterization of SHED. (a) Colony forming unit-fibroblasts (CFU-F) forming analysis is performed as described in the Methods. A representative image of CFU-F is demonstrated by toluidine blue staining. (b) Cell proliferation activity is examined by bromodeoxyuridine (BrdU) incorporation assay. A representative image of SHED with BrdU-positive neucleus. (c) Population doubling (PD) assay is performed until SHED loose the proliferative activity. (d) Telomerase activity in SHED is analyzed by telomeric repeat amplification protocol-quantitative polymerase reaction (TRAP-qPCR). 293, HKT293 cells; H.I., heat inactivated pretreatment. n = 3 for all groups. *** $P < 0.005$. Graph bars show the means ± SEM. (e) Flow cytometric analysis shows the expression of cell surface markers on SHED. Red histogram, staining with antibodies against target cell surface antigens; black histogram, isotype-matched antibody staining. PE, Phycoerythin. (f-h) Multipotent assays. Osteogenic capacity is analyzed by Alizarin red-S staining after 4 weeks of the osteogenic induction (f). Chondrogenic capacity is analyzed after 3 weeks of the chondrogenic induction by Alcian blue staining (g). Adipogenic capacity is analyzed after 4 weeks of the adipogenic induction by Oil red-O staining (h). f-h: Bars = 10 mm (f), 50 µm (g, h).

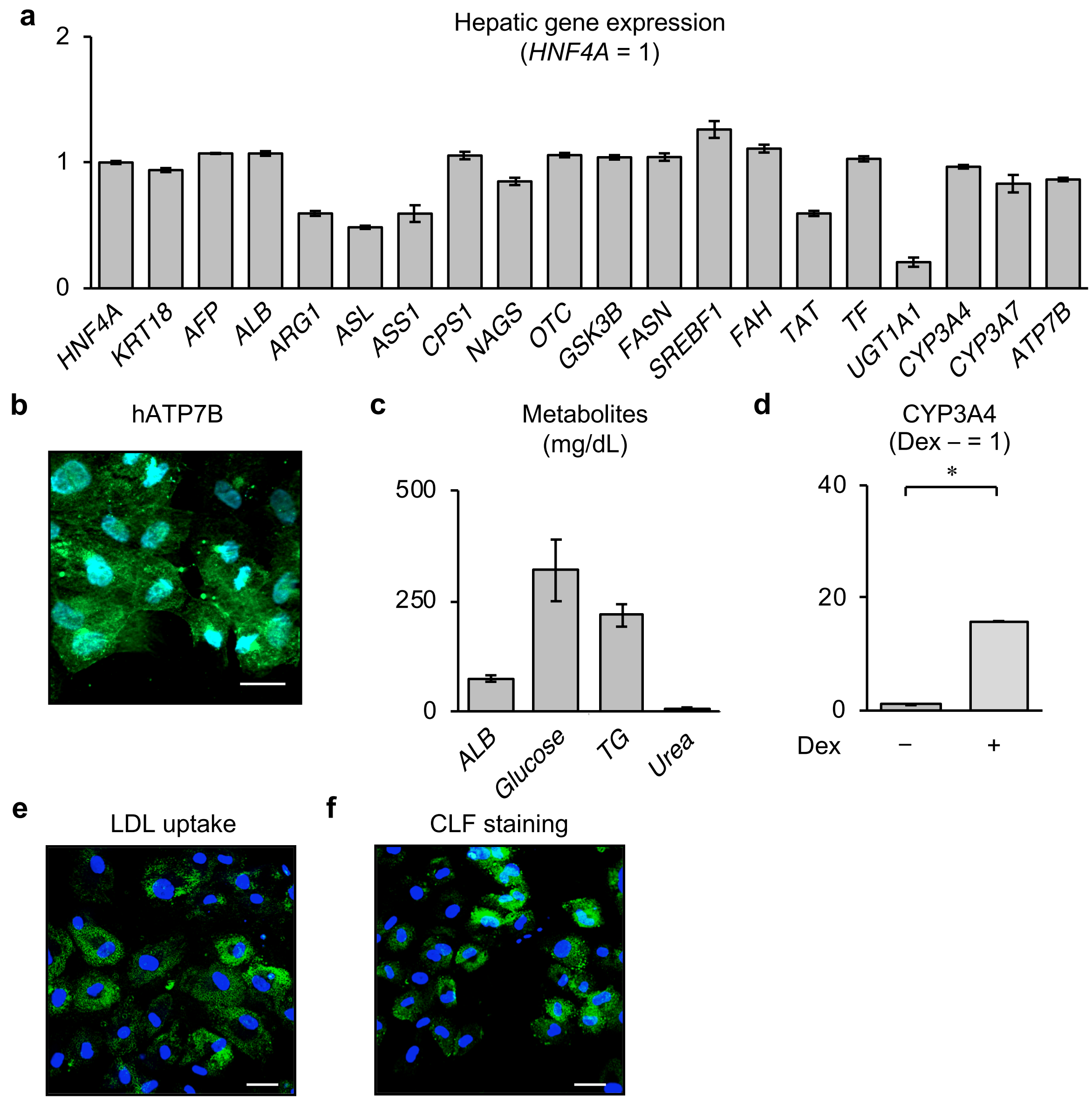

Supplementary Figure S2. Property of HepG2 cells. (a) Quantitative reverse transcription polymerase chain reaction (qRT-PCR) shows the expression of hepatocyte-specific genes in HepG2 cells. *ALB*, albumin; *ARG1*, arginase 1; *ASL,* argininosuccinate lyase; *ASS1,* argininosuccinate synthase 1; *ATP7B*, ATPase copper transporting beta; *CPS1,* carbamol-phosphate synthase 1; *CYP3A4,* cytochrome P450 family 3 subfamily A member 4; *CYP3A7,* cytochrome P450 family 3 subfamily A member 7; *FAH,* fumarylacetoacetate hydrolase; *FASN,* fatty acid synthase; *GSK3B*, glycogen synthase kinase 3 beta; *HNF4A*, hepatocyte nuclear factor 4 alpha; *KRT18*, keratin 18; *NAGS*, N-acetylglutamate synthase; *OTC*, ornithine carbamoyltransferase; *SREBF1*, sterol regulatory element binding transcription factor 1; *TAT*, tyrosine aminotransferase; *TF*, transferrin; *UGT1A1*, UDP glucuronosyltransferase family member A. The results are shown as the ratio to the expression of *HNF4A*. (b) Immunofluorescent analysis shows the expression of human ATP7B (hATP7B) in HepG2 cells. Nuclei are stained with 4',6-diamidino-2-phenylindole (DAPI). (c-f) Hepatic function assays in HepG2 cells are performed as described in the Methods. ALB, Albumin; TG, triglyceride (c). Activity of cytochrome P450 3A4 (CYP3A4) in HepG2 cells is measured treated in the presence or absence of dexamethasone (Dex; 50 μM). The results were shown as the ratio to the CYP3A4 activity in dexamethasone-untreated SHED-Heps. *** $P < 0.005$ (d). A representative image of low density lipoprotein (LDL) uptake in HepG2 cells is analyzed by DiI-Ac-LDL staining. (e). A representative image of bile acid transport in HepG2 cells is analyzed by cholyl-lysyl-fluorescein (CLF) staining (f). a, c, d: n = 3 for all groups. Graph bars show the means ± SEM. b, e, f: Bars = 30 μm.

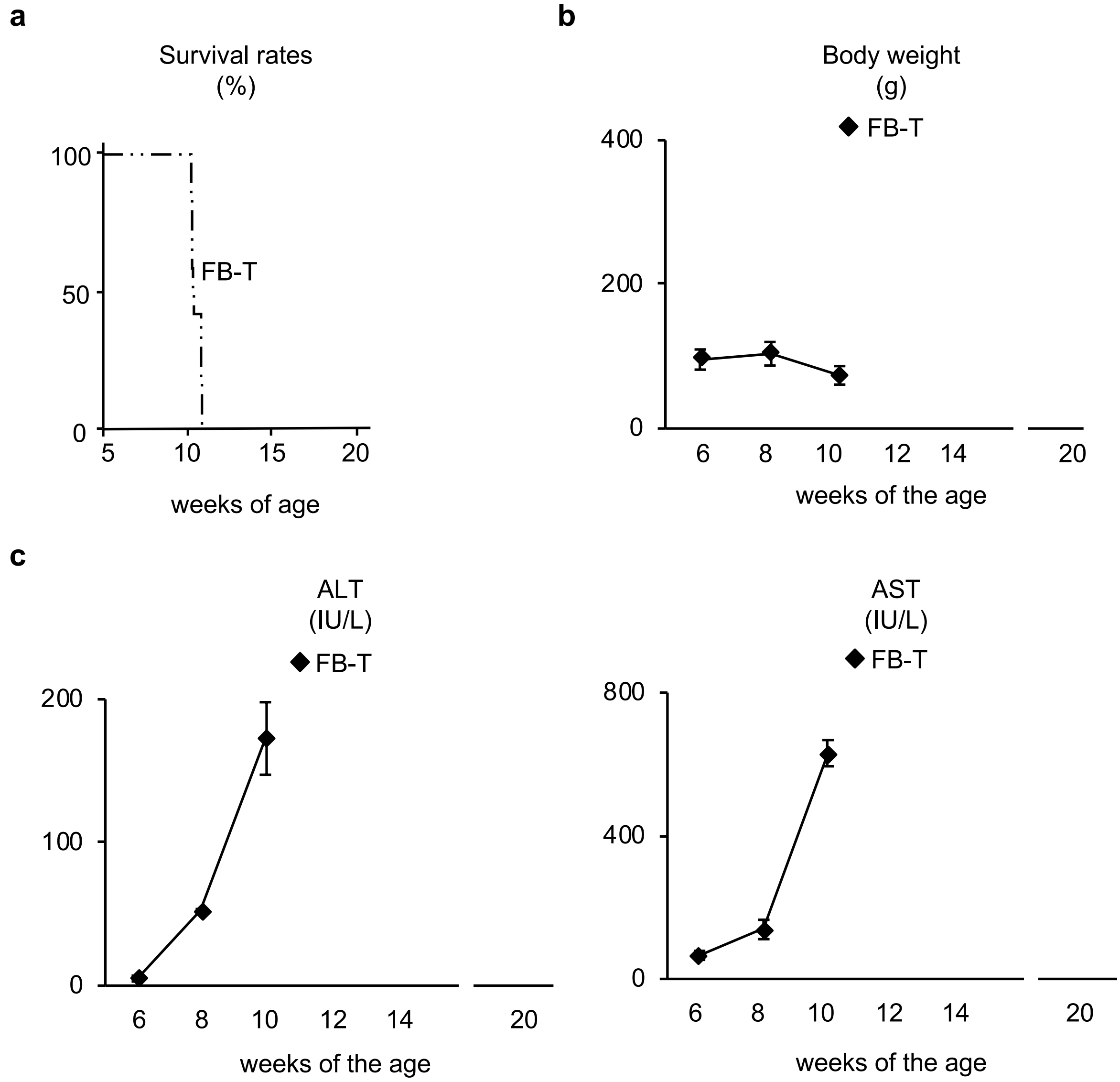

Supplementary Figure S3. Effects of human fibroblast (FB) transplantation on body weight and hepatic function of fulminant LEC rats. FBs are transplanted in copper-overloaded LEC rats at 6 weeks of the age. (a) Survival of the FB-transplanted fulminant LEC rats (FB-T, n = 5) is assayed by Kaplan-Meier curve. (b) The body weight of are measured at the indicated age. (c) Biochemical assay shows the serum levels of alanine transaminase (ALT) and aspartate aminotransferase (AST) of FB-transplanted fulminant LEC rats at the indicated age. b, c: n = 3 for all groups. Graph shows the means ± SD. b, c: black diamonds, FB-transplanted fulminant LEC rats (FB-T)

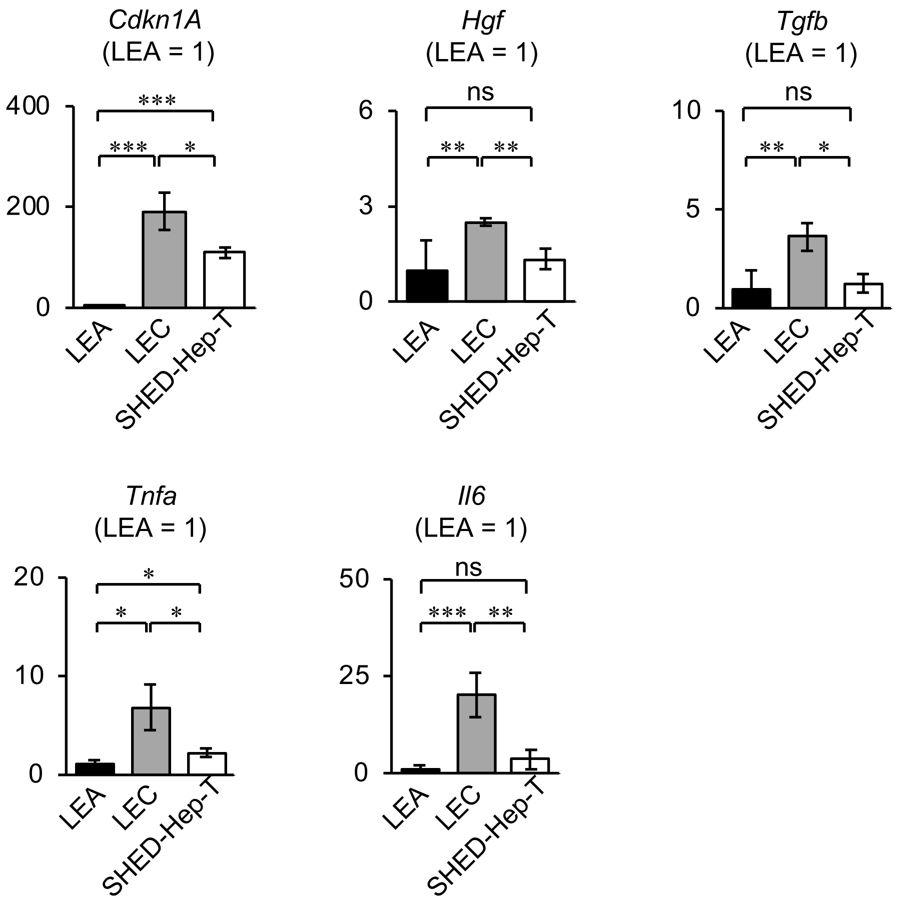

Supplementary Figure S4. Suppressive effect of SHED-Hep transplantation on hepatic failure in fulminant LEC rats. SHED-Heps are transplanted in copper-overloaded LEC rats at 6 weeks of the age. QRT-PCR assay demonstrates the expression of fulminant hepatitis-associated genes in the liver tissues after 4 weeks of SHED-Hep-transplantation in fulminant LEC rats. *Cdkn1a*, cyclin dependent kinase inhibitor 1A; *Hgf*, hepatocyte growth factor; *Il-6*, interleukin 6; *Tgfb,* transforming growth factor beta; *Tnfa,* tumor necrosis factor alpha. LEA, control LEA rats; LEC, non-transplanted fulminant LEC rats; SHED-Hep-T, SHED-Hep-transplanted fulminant LEC rats. n = 3 for all groups. * P < 0.05, ** P < 0.01, and *** P < 0.005. ns, no significance. The results are shown as the ratio to the expression of each gene in control LEA rats. Graph bars show the means ± SD.

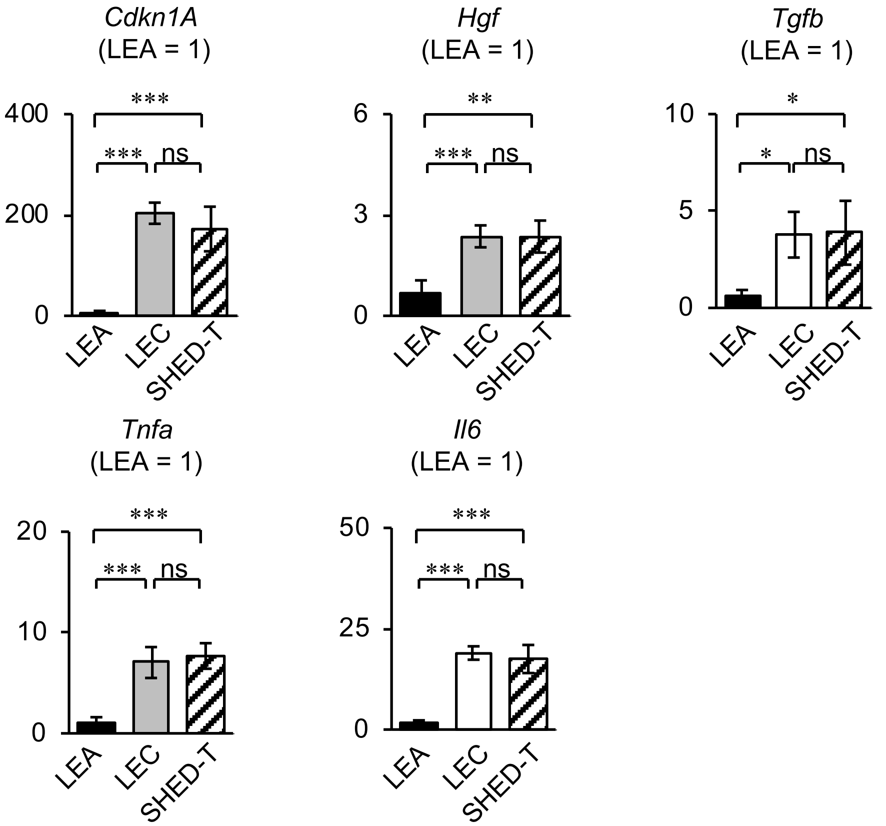

Supplementary Figure S5. Suppressive effect of SHED transplantation on hepatic failure in fulminant LEC rats. SHED are transplanted in copper-overloaded LEC rats at 6 weeks of the age. QRT-PCR assay demonstrates the expression of fulminant hepatitis-associated genes in the liver tissues after 4 weeks of SHED-transplantation in fulminant LEC rats. *Cdkn1a*, cyclin dependent kinase inhibitor 1A; *Hgf*, hepatocyte growth factor; *Il-6*, interleukin 6; *Tgfb,* transforming growth factor beta; *Tnfa,* tumor necrosis factor alpha. LEA, control LEA rats; LEC, non-transplanted fulminant LEC rats; SHED-T, SHED-transplanted fulminant LEC rats. n = 3 for all groups. $*$ $P < 0.05$, $**$ $P < 0.01$, and $***$ $P < 0.005$. ns, no significance. The results are shown as the ratio to the expression of each gene in control LEA rats. Graph bars show the means ± SD.

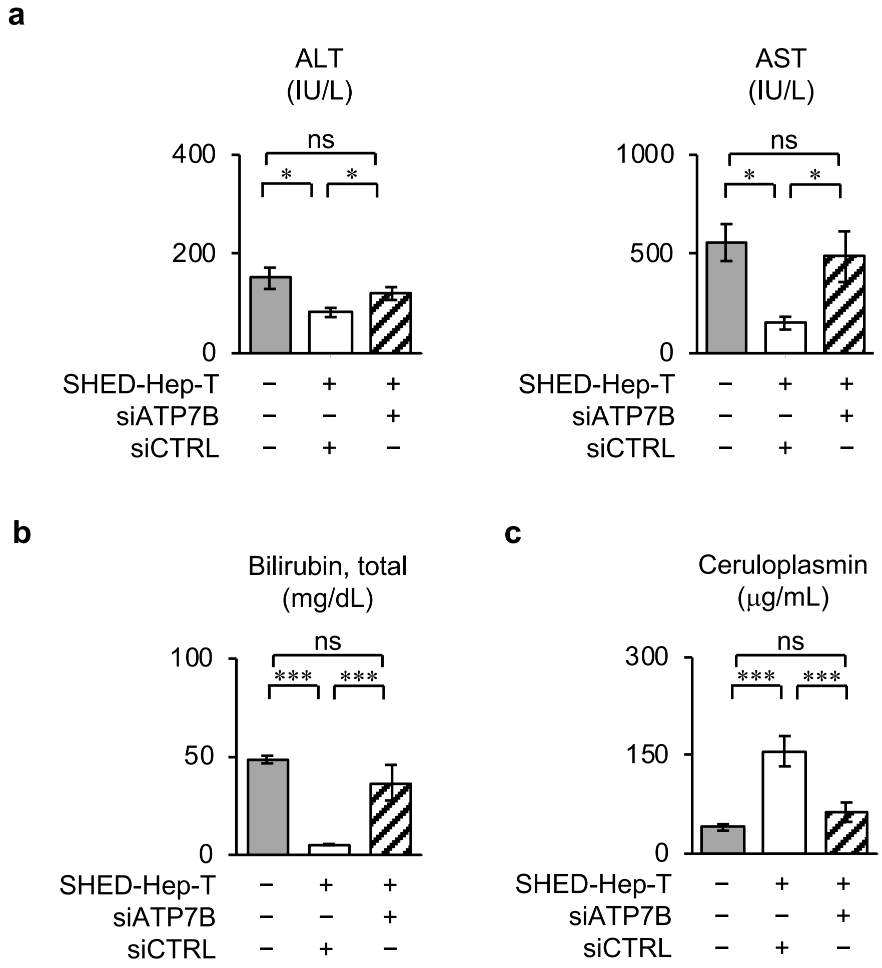

Supplementary Figure S6. Effect of ATP7B-knock-dowened SHED-Hep-transplantation on hepatic failure in fulminant LEC rats. ATP7B siRNA pre-treated SHED-Heps are transplanted in copper-overloaded LEC rats. Biochemical analysis and enzyme-labeled immunosorbent assay (ELISA) show the serum levels of alanine transaminase (ALT) and aspartate aminotransferase (AST) (a), total bilirubin (b), and celuroplasmin (c) in fulminant LEC rats at 10 weeks of the age after 4 weeks of the transplantation. SHED-Hep-T, SHED-Hep-transplantation; siATP7B, ATP7B siRNA pre-treatment; siCTRL, control siRNA pre-treatment. n = 3 for all groups. ∗ $P < 0.05$ and ∗∗∗ $P < 0.005$. ns, no significance. Graph bars show the means ± SD.

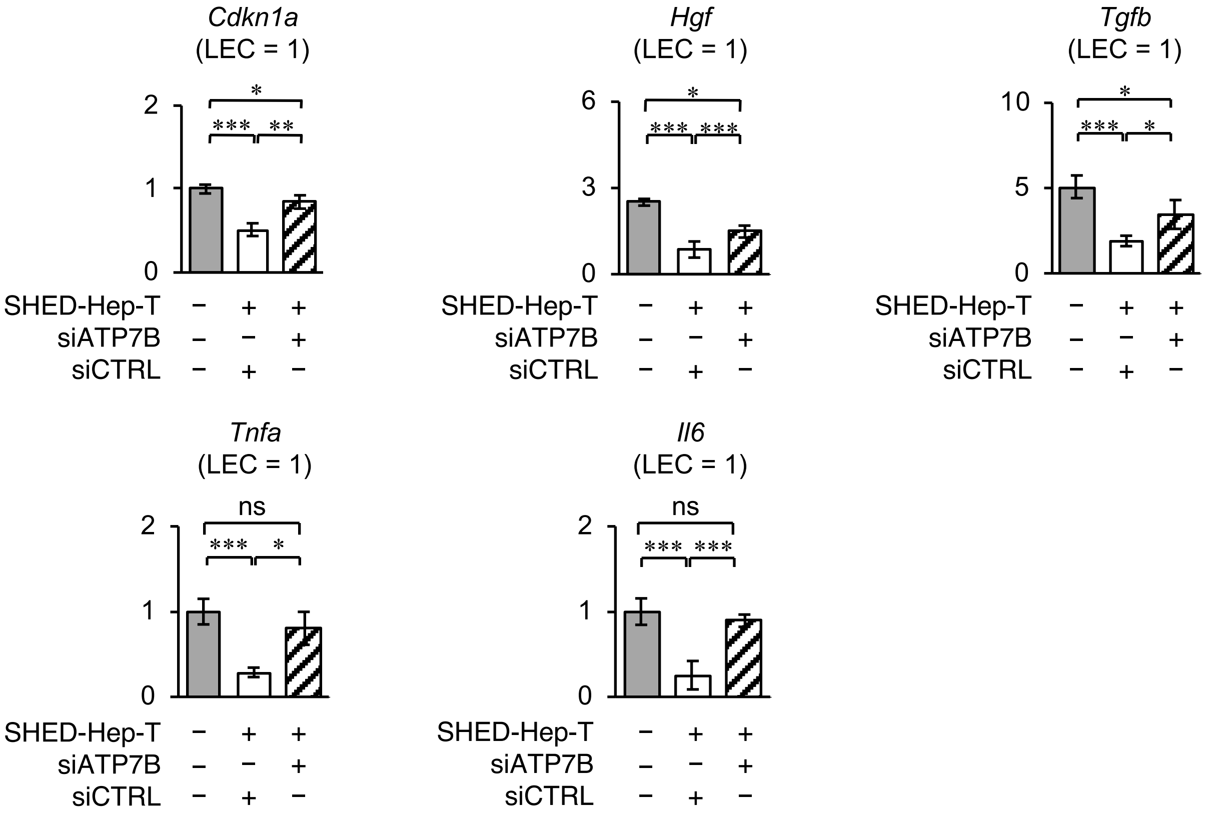

Supplementary Figure S7. Effects of ATP7B-knock-dowened SHED-Hep-transplantation on hepatic failure in fulminant LEC rats. ATP7B siRNA pre-treated SHED-Heps are transplanted in copper-overloaded LEC rats. QRT-PCR shows that expression hepatitis-associated in the liver tissues of fulminant LEC rats after 4 weeks of the transplantation. *Cdkn1a*, cyclin dependent kinase inhibitor 1A; *Hgf*, hepatocyte growth factor; *Il-6*, interleukin 6; *Tgfb,* transforming growth factor beta; *Tnfa,* tumor necrosis factor alpha. SHED-Hep-T, SHED-Hep-transplantation; siATP7B, ATP7B siRNA pre-treatment; siCTRL, control siRNA pre-treatment. n = 3 for all groups. * $P < 0.05$, ** $P < 0.01$, and *** $P< 0.005$. ns, no significance. The results are shown as the ratio to the expression of each gene in LEC rats without cell transplantation (LEC). Graph bars show the means ± SD.

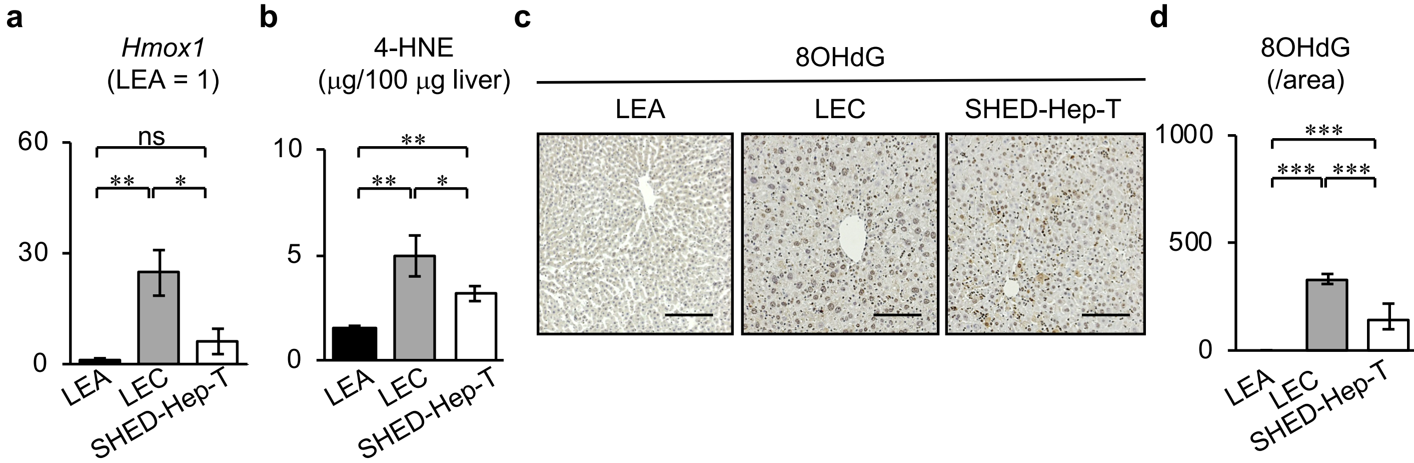

Supplementary Figure S8. SHED-Hep-transplantation suppresses hepatic oxidative injury in fulminant LEC rats. SHED-Heps are transplanted in copper-overloaded LEC rats at 6 weeks of the age. (a) QRT-PCR assay demonstrates the expression of heme oxidase 1 gene (*Hmox1*) in the liver tissues after 4 weeks of the transplantation. The results are shown as the ratio to the expression in control LEA rats. (b) Immunological assay shows the level of 4-hydroxy-2-deoxyguanosine (4-HNE) protein in liver tissues after 4 weeks of the transplantation. (c, d) Immunohistochemical assay demonstrates that the expression of 8-hydroxy-2‘-deoxyguanosine (8-OHdG) in liver tissues after 4 weeks of the transplantation. Nuclei are stained with hematoxylin. Representative images show the localization of 8-OHdG in the recipient liver tissue. Bars = 100 μm (c). Number of 8-OHdG-positive nuclei in the recipient liver tissue were counted (d). a-d: LEA, control LEA rats; LEC, non-transplanted LEC rats; SHED-Hep-T, SHED-Hep-transplanted LEC rats. a, b, d: n = 3 for all groups. ∗ $P < 0.05$, ∗∗ $P < 0.01$, and ∗∗∗ $P < 0.005$. ns: no significance. Graph bars show the means ± SD.

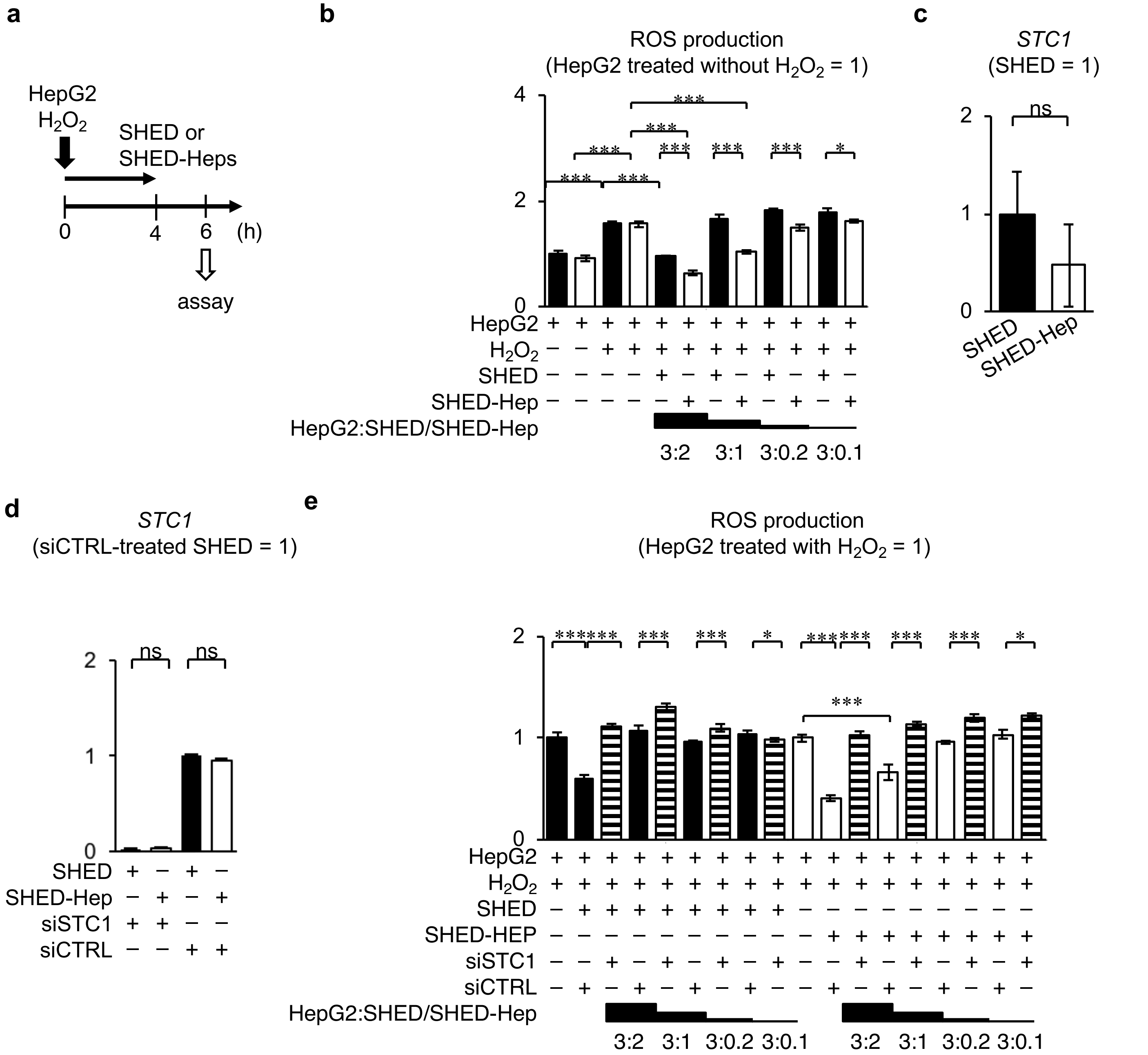

Supplementary Figure S9. SHED-Heps suppress ROS production from HepG2. Suppressive effect of SHED and SHED-Heps on the production of reactive oxidative species (ROS) under a coculture with HepG2 cells. (a) A schematic illustration of a coculture of HepG2 cells with SHED or SHED-Heps. SHED and SHED-Heps are co-cultured with HepG2 cells at different ratios under a stimulation with hydrogen oxide (H_2O_2; 25 μM) as described in the Methods. (b) The levels of ROS are measured as described in the Methods. (c) QRT-PCR demonstrates the expression of stanniocalcin 1 gene (*STC1*) in SHED and SHED-Heps. The results are shown as the ratio to the expression in SHED. (d) QRT-PCR demonstrates the effects of STC1 siRNA treatment on the *STC1* expression in SHED and SHED-Heps. The results are shown as the ratio to the expression in control siRNA-treated SHED. (e) Measurement of ROS production are measured as described in the Methods. b-e: n = 3 for all groups. ∗ $P < 0.05$ and ∗∗∗ $P < 0.005$. ns: no significance. Graph bars show the means ± SEM. b, e: The results are shown as the ratio to the expression of ROS in HepG2 treated without H_2O_2 (b) and HepG2 treated with H_2O_2 (e). c, d: The results are shown as the ratio to the expression of *STC1* in SHED (c) and siCTRL-treated SHED (d). d, e: siSTC1, STC1 siRNA pre-treatment; siCTRL, scrambled control siRNA pre-treatment.

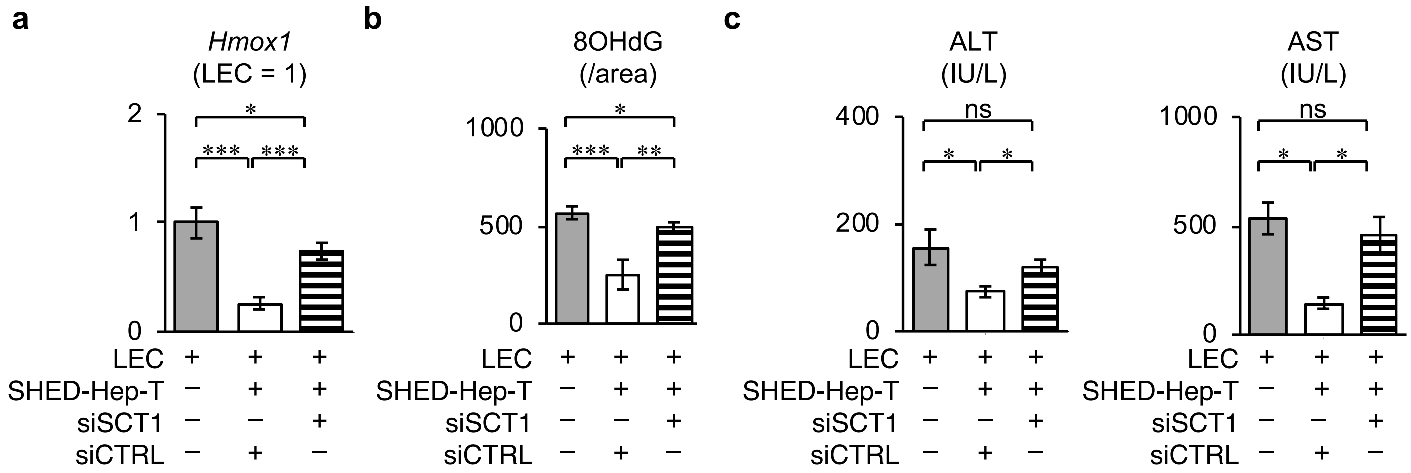

Supplementary Figure S10. Effects of SCT1-knock-dowened-SHED-Hep-transplantation on hepatic oxidative injury in fulminant LEC rats. STC1 siRNA pre-treated SHED-Heps are transplanted in copper-overloaded LEC rats at 6 weeks of the age. (a) QT-RT-PCR assay shows the expression of *Hmox1* in liver tissues after 4 weeks of the transplantation. The results are shown as the ratio to the expression in LEC rats without cell transplantation (LEC). (b) Immunohistochemical assay shows the number of 8-OHdG-positive nuclei in liver tissues after 4 weeks of the transplantation. (c) Biochemical assay shows the serum levels of ALT and AST after 4 weeks of the transplantation. SHED-Hep-T, SHED-Hep-transplantation; siSTC1, STC1 siRNA pre-treatment; siCTRL: scrambled control siRNA pre-treatment. a-c: n = 3 for all groups. $*$ $P < 0.05$, $**$ $P < 0.01$, and $***$ $P < 0.005$. ns, no significance. Graph bars show the means ± SD.
